# StAR-Related Lipid Transfer (START) Domains Across the Rice Pangenome Reveal How Ontogeny Recapitulated Selection Pressures During Rice Domestication

**DOI:** 10.3389/fgene.2021.737194

**Published:** 2021-09-08

**Authors:** Sanjeet Kumar Mahtha, Ravi Kiran Purama, Gitanjali Yadav

**Affiliations:** ^1^Computational Biology Laboratory, National Institute of Plant Genome Research, New Delhi, India; ^2^Department of Plant Sciences, University of Cambridge, Cambridge, United Kingdom

**Keywords:** genome-wide identification, gene duplication, synteny, START domain, *Oryza* species, gene expression, homeodomains

## Abstract

The StAR-related lipid transfer (START) domain containing proteins or START proteins, encoded by a plant amplified family of evolutionary conserved genes, play important roles in lipid binding, transport, signaling, and modulation of transcriptional activity in the plant kingdom, but there is limited information on their evolution, duplication, and associated sub- or neo-functionalization. Here we perform a comprehensive investigation of this family across the rice pangenome, using 10 wild and cultivated varieties. Conservation of START domains across all 10 rice genomes suggests low dispensability and critical functional roles for this family, further supported by chromosomal mapping, duplication and domain structure patterns. Analysis of synteny highlights a preponderance of segmental and dispersed duplication among STARTs, while transcriptomic investigation of the main cultivated variety *Oryza sativa* var. *japonica* reveals sub-functionalization amongst genes family members in terms of preferential expression across various developmental stages and anatomical parts, such as flowering. Ka/Ks ratios confirmed strong negative/purifying selection on START family evolution, implying that ontogeny recapitulated selection pressures during rice domestication. Our findings provide evidence for high conservation of START genes across rice varieties in numbers, as well as in their stringent regulation of Ka/Ks ratio, and showed strong functional dependency of plants on START proteins for their growth and reproductive development. We believe that our findings advance the limited knowledge about plant START domain diversity and evolution, and pave the way for more detailed assessment of individual structural classes of START proteins among plants and their domain specific substrate preferences, to complement existing studies in animals and yeast.

## Introduction

The steroidogenic acute regulatory protein (StAR) related lipid transfer (START) domain was initially identified and named after mammalian StAR protein of 30 kDa, which binds to cholesterol (Stocco, 2001). START domains are evolutionarily conserved domains of approximately 200–210 amino acids (Tsujishita and Hurley, 2000) and play a crucial role in the transfer of lipids/sterols, lipid signaling, and modulation of transcription activity (Ponting and Aravind, 1999; Soccio and Breslow, 2003). Presence of START domains across evolutionarily distant organisms indicates a conserved mechanism for protein-lipid/sterol interaction through hydrophobic pockets (Iyer et al., 2001). Interestingly, START domains are abundant in plants and often associated with homeodomain (HD) transcription factors, a feature unique to the plant kingdom (Schrick et al., 2004). For instance, 21 of the 90 HD family members identified in *Arabidopsis* possess START domains along with putative leucine zippers (Riechmann, 2002). Of these 21, 5 are from class III HD-ZIP subfamily and 16 are from class IV HD-ZIP subfamily (Schrick et al., 2004; Ariel et al., 2007).

The five genes from the class III HD-ZIP subfamily, namely PHB (phabulosa), PHV (phavoluta), REV (revoluta), CAN (corona) and ATHB8, have multiple and partially overlapping roles in development, including vasculature, organ polarity, and embryonic patterning of the shoot meristem (Prigge et al., 2005). In contrast, several members of the class IV HD-ZIP subfamily have roles in layer specific cell differentiation. ATML1 (*Arabidopsis thaliana* meristem layer 1) and PDF2 (protodermal factor 2) have putative roles in epidermal differentiation (Lu et al., 1996; Abe et al., 2003). GL2 (glabra 2) is required for the differentiation of epidermal cells in the shoot (Rerie et al., 1994), root (Di Cristina et al., 1996), and seed (Western et al., 2001). ROC1 (rice outer most cell-specific gene 1) of rice has similar function as ATML1, where its expression is limited to the outermost epidermal layer from the early embryogenesis (Ito et al., 2002). OSTF1 (*Oryza sativa* transcription factor 1) also preferentially expressed in epidermis, and developmentally regulated during early embryogenesis (Yang et al., 2002).

Since HD START proteins act as transcription factors in plants, a major expectation is that START, when it binds to sterol, regulates gene expression similar to steroid hormone receptors from animals, this mechanism would allow cell differentiation to be linked with lipid metabolism in plants (Ponting and Aravind, 1999; Venkata and Schirck, 2006). Plant START domains were shown to be required for transcription factor activity in class IV HD-ZIP protein “GL2” in *Arabidopsis*, and they were also found to have ligand-binding modules, similar to mammalian START domains (Schrick et al., 2014). Activated expression of HDG11 START domain confers drought tolerance with reduced stomatal density and improved root system in *Arabidopsis* (Yu et al., 2008).

Although START domains are amplified in plants and appear to have diverse functions, a thorough knowledge of the mechanism of amplification and gene duplication in this family is lacking. With the availability of many varietal genomes, and huge genotypic variation ranging from diploids to polyploids, *Oryza* has a long history of use as a model monocot food crop. Furthermore, with a wide evolutionary history that spans more than 15 million years, *Oryza* is an ideal prototype for such a study (Chatterjee, 1947; Li et al., 2014; Stein et al., 2018). Rice also has a major social significance, consumed by half the global population with an estimated 20% of human dietary calories that are met only by the domesticated Asian rice variety, thereby making it a target for improvement toward addressing the food security issue of a growing world population under a changing climate.

In this work, we focus on 10 diploid *Oryza* species, including three cultivated varieties having AA genotype, *O. sativa* var. *indica*, *O. sativa* var. *japonica* (Asian cultivated variety), along with *Oryza glaberrima (African cultivated variety).* Five of the seven wild *Oryza* species included in this analysis have AA genotype, namely *Oryza rufipogon, Oryza nivara, Oryza barthii, Oryza glumaepatula*, and *Oryza meridionalis*, while two others have BB and FF genotypes, *Oryza punctata* and *Oryza brachyantha*, respectively. Regardless of genotypes, all 10 species have enormous repeats, varying from one fourth to half the genome size (Stein et al., 2018). In general, repeat regions are accumulated with increasing evolutionary order from early-evolved wild relatives such as *O. brachyantha* and *O. meridionalis* (approximately 27–29%) to the recent cultivated varieties *O. sativa* var. *japonica* and *indica* (approximately 40–50%). *O. punctata* is an exception, despite being an early evolved wild species, has half of its genome containing repeats; resulting in a huge repertoire of synteny within the *Oryza* genome, varying from 90 to 97% (Stein et al., 2018). In addition, there is a gene flow among AA type *Oryza* genomes, which needs to be thoroughly investigated to understand the specific changes that occurred in the gene families (Li et al., 2014; Stein et al., 2018). The expanded gene family of START domains can be single or multi-domain (Schrick et al., 2004; Alpy and Tomasetto, 2005), and has been reported to associate with several other domains such as homeodomain, MEKHLA, and PH (pleckstrin homology) domains, known for their involvement in transcription regulation, sensing and signaling, respectively (Ponting and Aravind, 1999; Schrick et al., 2004; Mukherjee and Bürglin, 2006; Venkata and Schirck, 2006). Among the multi-domain START proteins, ligand binding by the START domain can modulate the activity of other domains that co-occur with START domains (Ponting and Aravind, 1999; Iyer et al., 2001; Schrick et al., 2014).

In this article, we aim to provide a comprehensive comparative genomic analysis of START genes across the 10 *Oryza* genomes, investigated all the way from identification and classification to sequence homology, genome-wide mapping, and duplication analysis of START genes. Available transcriptomic data for *O. sativa* var. *japonica* was investigated to understand co-expression patterns for potential sub- or neo-functionalization among these genes. Genome wide identification revealed a total of 249 START genes taking all 10 rice species together and showed that the gene family size for START genes varies from 22 to 28. Domain structure analysis (DSA) confirmed the presence of additional functional domains associated with STARTs such as HDs, MEKHLA, PH, and DUF1336 and classified the START proteins into total eight unique combinations based on associated domain patterns. Phylogenetics revealed the extent of divergence amongst START proteins and we find distinct clusters based on above-mentioned domain structure patterns. The genome-wide mapping showed that these genes are distributed among 11 chromosomes out of 12 in most of the cultivated and wild rice species. Gene duplication studies indicate that START genes preferred segmental and dispersed modes of duplication for gene expansion under natural selection. Hierarchical clustering of transcriptome data revealed many duplicated gene pairs have similar expression patterns across developmental stages and anatomy. In summary, this is comparative genomics of START genes across wild and cultivated rice and enhances our understanding of the mechanism of START gene amplification in plants.

## Materials and Methods

### Data Collection

The complete genomic sequences, protein sequences, and annotation information of nine species of *Oryza*, including seven wild varieties *O. brachyantha* (*Obra*_*w*_), *O. punctata* (*Opun*_*w*_), *O. meridionalis* (*Omer*_*w*_), *O. glumaepatula* (*Oglu*_*w*_), *O. barthii* (*Obar*_*w*_), *O. nivara* (*Oniv*_*w*_), and *O. rufipogon* (*Orup*_*w*_) along with two cultivated varieties *O. glaberrima* (*Ogla*_*c*_) and *O. sativa* var. *indica* (*Oind*_*c*_), were downloaded from Ensembl (Kersey et al., 2018). In addition, similar data for the main cultivated variety, *O. sativa* var. *japonica* (*Ojap*_*c*_) was downloaded from the Phytozome v12 having the latest updated version of sequences (Goodstein et al., 2011). Throughout this work, these 10 species are referred to in subscripted ***Oabc*_*x*_** format where *abc* represents first three letters of the species/subspecies name, while the subscript “*x*” is *c or w*, representing cultivated or wild nature, respectively.

### Identification and Validation of START Proteins

Previously reviewed and characterized sequences of 109 START domain-containing proteins were collected from InterPro consortium (Jones et al., 2014). The START regions in these proteins were extracted based on annotated border residues, and sequence redundancy was removed at cut off 95% using CD-hit (Huang et al., 2010). The resulting 84 sequences were used to construct a profile Hidden Markov Model (HMM) with HMMER 3.2.1^1^ (Eddy, 1998; Finn et al., 2011). The profile was run against all 10 *Oryza* proteomes and short hits (sequence length <100 residues) were discarded, followed by removal of redundancy, performed by filtering out all but the longest peptide for each protein. The validation of identified hits as START family proteins was performed using Conserved Domain Database (CDD) (Marchler-Bauer et al., 2014).

### Domain Structure Analysis of START Domain Containing Proteins

The putative START domain containing proteins identified as described above were subjected to domain structural pattern analysis to ascertain additional domains associated with START. DSA was carried out using a web-based Batch CD-search Tool, selecting CDD (Marchler-Bauer et al., 2011). CDD includes curated data from NCBI (National Center for Biotechnology Information) (Agarwala et al., 2017) SMART (Simple Modular Architecture Research Tool) (Letunic et al., 2015) Pfam (protein families) database (Finn et al., 2014), PRK [PRotein K(c)lusters] (Maglott et al., 2011), COG (Clusters of Orthologous Groups of proteins) (Tatusov et al., 2003), and TIGRFAMs (The Institute for Genomic Research’s database of protein families) (Haft et al., 2003). The additional associated domains, as identified in this step were used to classify rice START domains into various domain structural classes. Besides, transmembrane helical segments associated with START domains were predicted using TMHMM Server v. 2.0 (Krogh et al., 2001). The domain arrangement of START proteins was illustrated using IBS v.1.0 (Liu et al., 2015).

### Gene Structure Analysis of START Coding Genes

Gene structure analysis (GSA) was carried out to understand the exon–intron patterns for different classes of START encoding genes among 10 rice species. Gene structure was visualized using Gene Structure Display Server (GDSD) (Hu et al., 2015). The corresponding Gene and CoDing Sequence (CDS) of each START encoding protein were used as input for GSA. Visualizing the structure and annotated features of genes can help in understanding function and evolution intuitively. The visualization of gene features such as composition and position of exons and introns for genes offers visual presentation for integrating annotation for each conserved domain. Accordingly, we highlighted the exons coding for different types of functional domains across START proteins, which further enabled us to understand exon–intron pattern across wild and cultivated rice genomes.

### Genome-Wide Mapping and Identification of Homologous and Orthologous START Coding Genes Amongst 10 *Oryza* Species

In order to map the START coding genes onto rice chromosomes, gene location data was extracted from the respective GFF annotation files (general feature format), and karyotype information was extracted based on chromosomal length. Chromosomal visualization of genes in all 10 rice species was done using Circos (Krzywinski et al., 2009), colored by structural class. Orthologous START genes in nine *Oryza* species were identified in reference to *Ojap*_*c*_ by local protein BLAST, based on maximum identity and similarity.

### Phylogenetic Analysis of Different Structural Classes of START Domain Containing Proteins

Phylogenetic analysis was carried out for different structural classes of START proteins across all 10 species, to explore intra- and inter-species divergence. All 249 full-length START proteins in the 10 *Oryza* genomes and 35 sequences from *A. thaliana* were included in the phylogenetic study. The available gene symbols are used in case of *O. sativa* var. *japonica* and *A. thaliana.* Multiple sequence alignment was performed using MUSCLE at default settings (Madeira et al., 2019). Aligned sequences were used for phylogenetic tree construction. The tree was generated through RAxML (raxmlGUI *v*2.0.5) (Stamatakis, 2014; Edler et al., 2021) using maximum likelihood method at bootstrap value of 1000 and the tree was visualized using Figtree v1.4.2 (Rambaut, 2014).^2^

### Gene Duplications, Collinearity, and Nucleotide Substitution Rates

The MCScanX software package (Wang et al., 2012) was used to identify various duplication modes for START genes among *Oryza* species. This program works on the all-vs-all BLASTp results and this was performed for all 10 rice proteomes (Altschul et al., 1990). The results were fed into duplicate gene classifier, a module of MCScanX, to detect dispersed, proximal, tandem, and/or segmental duplications. The criteria used by the duplicate gene classifier for assignment of duplication modes were as follows: Initially, all genes were ranked in order of occurrence along the chromosome and labeled as singletons. Gene pairs were evaluated based on BLASTp hits, and pairs identified at a cut-off distance of 20 were re-labeled as “dispersed duplicates.” Gene pairs that showed gene rank difference of less than 20 were re-labeled as “proximal duplicates” while the gene pairs found next to each other (i.e., gene rank difference = 1), were re-labeled as “tandem duplicates.” Following this, collinear blocks within the individual plant genomes were identified, and anchor genes found in collinear blocks were re-labeled as “segmental/WGD duplicates.” Finally, all genes were assigned to different duplication modes based on the following order of priority, i.e., whole genome duplication (WGD) / segmental > tandem > proximal > dispersed. Unduplicated genes (that occur only once in the genome) retained their original classification as “singletons” (Wang et al., 2012). Collinear blocks for all proteins within individual genomes were generated by MCScanX module (gray color links). START gene homologs within collinear blocks were highlighted using the previously described domain structure class colors. MCScanX-transposed (Wang et al., 2013) was used to find the newly trans-located START homologs from their original ancestral locations to a novel locus in *Ojap*_*c*_. The START gene homologs obtained from the interspecies BLASTp between *Ojap*_*c*_ and the other nine *Oryza* genomes were analyzed for non-synonymous (Ka) and synonymous (Ks) substitution rates by KaKs calculator 2.0 (Wang et al., 2010).

### Transcriptome Analysis and Hierarchical Clustering

Gene expression levels of 28 START genes in the major globally cultivated rice variety *Ojap*_*c*_ were investigated using RNA-seq data “Os_mRNAseq_Rice_GL-0” (MSU v7.0) on the Genevestigator platform (Hruz et al., 2008). The conditional search tool was used to analyze gene expression across nine developmental stages and 13 anatomical parts, and their log-transformed values were further arranged in hierarchical clustering groups based on Pearson correlation coefficients of START genes by selecting optimal leaf ordering for both, developmental stages and anatomical parts. The heatmaps were generated using Mev_v4.8 (Saeed et al., 2003).

## Results

### Identification of START Genes Amongst Wild and Cultivated Varieties of Rice

The HMM profiles, constructed based on known sequences, were used to perform the hmmsearch against 10 *Oryza* proteomes (listed in Table 1) and only those hits were retained that matched the minimum length criteria, and were validated for the presence of START, as described in section “Materials and Methods.” This led to the identification of 360 START proteins (including protein isoforms), coded by 249 gene transcripts across the 10 species of rice. In order to remove redundancy, only the single longest protein coded by each set of gene transcripts was retained for downstream analysis. START coding genes were found to vary from 22 to 28 in these *Oryza* species as shown in Table 1.

**TABLE 1:** Identification and domain structure analysis of START proteins across cultivated and wild rice species.

Name of plants and (genotype)	Species code	Total genes in each genome	No. of START genes	HZSM	SM	HZS	HS	PSD	PS	SD	mS	TM containing START proteins
Cultivated rice species												
*Oryza sativa* var. *japonica* (AA)	*Ojap*_*c*_	42,189	28	4	2	2	9	2		1	8	2
*Oryza sativa* var. *indica* (AA)	*Oind*_*c*_	42,031	27	5		2	9	3		1	7	2
*Oryza glaberrima* (AA)	*Ogla*_*c*_	34,130	24	4	1	2	9	1		2	5	1
Wild rice species												
*Oryza rufipogon* (AA)	*Oruf*_*w*_	37,912	25	5		2	9	2			7	2
*Oryza nivara* (AA)	*Oniv*_*w*_	37,026	26	5		2	9	4			6	2
*Oryza barthii* (AA)	*Obar*_*w*_	35,553	23	5		2	8	2			6	1
*Oryza glumaepatula* (AA)	*Oglu*_*w*_	36,379	25	5		2	9	3			6	2
*Oryza meridionalis* (AA)	*Omer*_*w*_	30,241	22	5		2	7	3			5	1
*Oryza punctata* (BB)	*Opun*_*w*_	32,550	24	6		2	10	2			4	2
*Oryza brachyantha*(FF)	*Obra*_*w*_	32,463	25	5		2	8	3	1	1	5	2
Total			249	49	3	20	87	25	1	5	59	17

*HZSM, HD bZIP START MEKHLA; SM, START MEKHLA; HZS, HD bZIP START; HS, HD START; PSD, PH START DUF1336; PS, PH START; SD, START DUF1336; mS, minimal START; TM, transmembrane segments. The species are first categorized based on their cultivated and wild nature and then ranked in the order of evolution, from recent to oldest evolved.*

As can be seen from Table 1, the most widely cultivated varieties *Ojap*_*c*_, and *Oind*_*c*_ possess the highest numbers of START coding genes, when compared to early evolved African cultivated species *Ogla*_*c*_ and the seven wild species. The oldest AA variety *Omer*_*w*_ has 22 START coding genes, lowest among all, although START numbers do not vary greatly between species, and their numbers are proportional to genome size in most cases. Among the wild varieties, the earliest evolved *Obra*_*w*_ has the same number of START genes as the most recently evolved wild variety *Oruf*_*w*_. The breakup of these START domains, in terms of potential functions based on domain combinations, is explored further in the next section, but these general numbers suggest that the increase in START domains among cultivated rices, may reflect an evolving role of STARTs in stress induction or stress response. For accession IDs of START coding genes found in all 10 species, along with protein and domain information, see Supplementary Table 1.

### Domain Structure Analysis and Classification of START Proteins

The DSA was carried out to find the additional domains associated with START domains and to understand their arrangement among the START proteins. START proteins have been known to exist both as minimal START domains, as well as in association with other functional domains, and this domain organization has been used as a criterion for their classification along with information on specific ligands, which they bind (Schrick et al., 2004; Alpy and Tomasetto, 2005). We explored the domain structure of all 249 identified START domains, and classified them into eight groups, as shown in Table 1, depending on the combinatorial patterns of STARTs with additional domains such as HD, bZIP (basic leucine zipper domain), MEKHLA domains, PH domain, and DUF1336 (domain of unknown function). Six of these eight groups have been reported earlier (Schrick et al., 2004, 2014), including (a) mS, i.e., minimal START lacking any additional domains, (b) HS (having HD), (c) HZS (containing HD and bZIP), (d) HZSM (having HD, bZIP, and MEKHLA), (e) PSD (with PH and DUF1336), and (f) SD (START with DUF1336), while two new combinations (not reported earlier) were also seen, namely (g) SM (i.e., START with MEKHLA) and (h) PS (START with PH). Interestingly, these last two combinations are the only ones that are either completely absent from the cultivated varieties (as in case of PS), or completely absent from wild varieties (as in case of SM).

As can be seen in Table 1, almost 24% of rice START domains belong to minimal START (i.e., lacking any additional domains), while homeodomains constitute the largest category of domains co-occurring with STARTs. The recently evolved cultivated rices (*Ojap*_*c*_ or *Oind_*c*_)* have a higher number of minimal STARTs compared to early evolved wild species *(Obra_*w*_* or *Opun_*w*_)*. We have previously shown that the HD associated with STARTs in plants has unique roles in plant transcription (Schrick et al., 2014), and this seems to be an ancient feature since all wild rice species also have the homeodomains. The HDs are always found in association with a leucine zipper in class III and class IV HD-zip family of plant START proteins. Over 60% of the 249 identified domains in rice have these homeodomains in combination with leucine zippers, which in turn, can be of two types; (a) class III HD ZIP START domains with a universally conserved basic leucine zipper known as bZIP, and (b) class IV HD ZIP STARTs, with a plant exclusive leucine zipper, known as ZLZ (Schrick et al., 2004; Ariel et al., 2007). Another domain, MEKHLA, often seen associated with the class III HD bZIP START proteins (Mukherjee and Bürglin, 2006), is completely missing from the START domains in all the wild rice species (SM family), as can be seen from Table 1. Our DSA methodology is based on CDD (Marchler-Bauer et al., 2014) which does not recognize the ZLZ, hence we use the term “HD-START” for class IV type proteins throughout this study.

**FIGURE 1:**
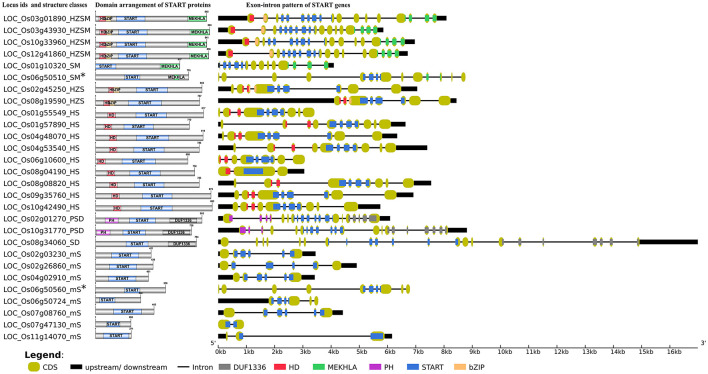
Schematic overview of domain arrangement of START proteins and gene structure of all 28 STARTs genes in *Oryza sativa* var. *japonica.* Index shows exonic colors for homeodomain (HD), homeodomain associated basic leucine zipper domain (HD bZIP), START domain, PH domain, domain of unidentified function (DUF1336), and MEKHLA. The exon portions that code the protein inter-domain regions (gray colored regions in domain arrangement) are depicted in mint shades at gene structure. (*Gene pairs are proximally duplicated and shows higher resemblance in gene structure).

Interestingly, the difference between domain structure of wild and cultivated rice does not appear to arise from the homeodomain containing STARTs, all of which occur in large numbers and with moderate uniformity across all rices (see Table 1). Apart from the HD containing START domains, the other two major domains that co-occur with STARTs are the PH at the N-terminus, and DUF1336 domains at the C-terminus. These form unusual combinations, two of which have been observed for the first time in this work, as mentioned earlier, and are starkly distinct between wild and cultivated rices; the dual combinations of START DUF1336 (five), START MEKHLA (three), and PH START (one). In fact, a START domain in combination with the PH alone, has only been observed in the earliest evolved wild rice namely, *Obra*_*w*_. Similarly, very few domains show the combination of START domain alone with DUF1336, but the triple combination (PSD category) is seen frequently (35% of non-HD START combinations) across all rices, suggesting that these three domains are more effective in combination, rather than alone. PH domains are well known for intracellular signaling or as constituents of the cytoskeleton proteins. This domain also binds with phosphatidylinositol within biological membranes, thus playing roles in membrane recruitment, subcellular targeting or enabling interactions with other components of the signal transduction pathways (Mayer et al., 1993; Ingley and Hemmings, 1994). Intriguingly, another connection to this role is evident in minimal STARTs, 30% of which were found to have transmembrane (TM) segments (17 in all; 11 with two TM segments and 6 with single TM), that shows a huge similarity to a specific class of mammalian STARTs, namely the phosphatidylcholine transfer proteins (STARD2/PCTP) that preferentially bind to phosphatidylcholine (Satheesh et al., 2016). That PH domains are present singly with START domains in the earliest known rice, and not elsewhere, as well as the presence of TM segments, but only in minimal STARTs, suggests that initiation of association with other domains began with membrane interfaces, and the addition of other, newer domains, may have been critical to the evolution of START functional diversity. The illustrative image for domain organization of 28 START proteins from *Ojap*_*c*_ is given in Figure 1. The detailed DSA report of 249 START proteins along with the domain sizes and positions is provided in Supplementary Table 1.

### Gene Structure Analysis of START Coding Genes

Gene structure analysis for all 249 START domains was performed as described in sections “Materials and Methods,” and “Results” are depicted in Table 2, listing exon numbers for each functional domain within and between the eight START categories described in the previous section. The GSA for main cultivated variety *Ojap*_*c*_ along with its full-length protein domains pattern is depicted in Figure 1, whereas full gene structure maps and complete exon–intron details of all START domains in all 10 rices are provided as Supplementary Tables 1, 2 and Supplementary Figures 1A–J. In general, gene sizes vary between 0.5 to 32 kb, with some of the minimal STARTs in the oldest wild rice *Obra*_*w*_ encoded by a single exon, while few PSD class STARTS in wild rices have up to 34 exons. The overall pattern (see Table 2) is that exon numbers for the START region itself are quite conserved within specific structural classes, and the variability between wild and cultivated rice stems from exon numbers of the associated domains in these proteins. Exon numbers are also highly variable across the eight classes of START domains, with the greatest variability reflected in the minimal STARTs which contain very large intronic regions and long lengths of upstream and downstream UTRs (up to 17 kb), suggesting a potential for the addition of new domains, exon creation and alternate splicing. Figure 1 reveals similarities between gene structure of the newly observed SM class of STARTs with other categories; GSA of one of the SM genes is almost identical with HZSM members, after losing the HZ fragment, whereas the other SM gene has a GSA identical to a member of the minimal STARTs (Figure 1), suggesting a gain of function. They are also proximally duplicated where SM classes showed higher expression in both anatomical part and development stages while mS classes are poorly expressed (see the section on gene expression). Both pairs of genes are on the same chromosomes, adding support to these hypotheses, as discussed in the subsequent sections on chromosomal mapping and phylogenetics.

**TABLE 2:** Gene structure analysis: number of exons involve in coding full-length START proteins for different structural classes amongst 10 cultivated and wild *Oryza* species.

Name of plants	HZSM	SM	HZS	HS	PSD	PS	SD	mS
**Cultivated rice species**								
*Ojap*_*c*_	18 or 19 (8)	14 (4 or 5)	9 (4)	4–12 (1 or 4)	20 or 22 (7 or 8)		23 (7)	2–10 (2–5)
*Oind*_*c*_	15–19 (7 or 8)		8 or 9 (4)	4–12 (1–5)	19–22 (7 or 8)		23 (7)	3–7 (2–5)
*Ogla*_*c*_	18 (8)	14 (6)	8 or 9 (4)	3–11 (1–4)	21 (7)		20 or 22 (7 or 8)	3 or 6 (2–5)
**Wild rice species**								
*Oruf*_*w*_	16–20 (7 or 8)		9 (4)	5–12 (1 or 4)	20 or 24 (5 or 7)			2–7 (2–5)
*Oniv*_*w*_	16–21 (7 or 8)		8 or 9 (4)	5–13 (1 or 4)	20–25 (5 or 7)			3–11(2–5)
*Obar*_*w*_	16–20 (7 or 8)		9 (4)	9–12 (3 or 4)	20 or 23 (5 or 7)			4–10 (3–5)
*Oglu*_*w*_	16–20 (7 or 8)		9 or 10 (4)	5–14 (1–5)	19–26 (2–7)			2–19 2–5)
*Omer*_*w*_	17–21 (7 or 8)		9 or 10 (4)	5–15 (1–4)	19–23 (2–5)			6–15 (4 or 5)
*Opun*_*w*_	15–20 (7 or 8)		9 (4)	5–12 (1–4)	19 or 34 (6 0r 8)			3–7 (2–5)
*Obra*_*w*_	18–20 (7 or 8)		11 (4)	5–13 (2–4)	20–24 (7 or 8)	12 (6)	23 (8)	1–8 (1–5)

*The values given in parentheses are the number of exons that code for START domains regions alone. Acronyms/codes same as Table 1.*

Apart from the above mentioned differences in the number of exons, START coding genes also vary in intron length and Untranslated Regions (UTRs) across the cultivated and wild rices. There is still much that is unknown about flanking UTRs at both the terminals of mRNA in the form of 3′-UTR and 5′-UTR, and although UTR regions have often been implicated in regulatory aspects of gene expression, they need to be investigated further. Almost one-third of all START coding genes reveal long sections of 3′-UTR and 5′-UTR (ranging from few nucleotides up to 17 kb), but the African and Asian cultivated rices (*Oind*_*c*_ and *Ogla*_*c*_) appear to completely lack these at both termini. Clearly, cultivated rices vary by ancestors, and this is reflected in their inherent genetic diversity, as observed between *Oind*_*c*_ and *Ojap*_*c.*_ As can be seen in Supplementary Figures 1A–J and Supplementary Table 2, UTR lengths were observed to be very long in minimal START genes, along with very long intron lengths, both features suggesting the potential for evolution *via* introduction of new function. Among the various classes of START genes, HZSM shows the shortest exon and introns regions while PSD and SD classes show distinctive combination of several exons and longer introns, aside from long stretches of UTR regions. Most classes have exons flanked by long introns but the HZSM and PSD have exons flanked by short introns. Cultivated rices having fewer cases of long flanking introns, further emphasize the greater genetic diversity in wild rices and scope for exon creation, alternate splicing and addition of functional features.

### Ortholog Analysis and Chromosomal Distribution

**FIGURE 2:**
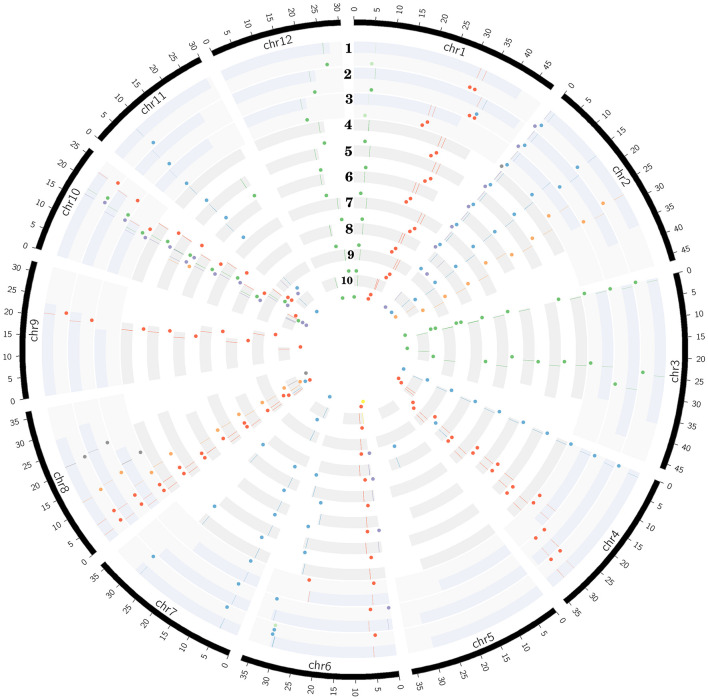
Distribution of all eight START categories (colored dots shown internal to chromosomes in circular ribbons and lines on chromosomes) across 10 *Oryza* genomes (each ribbon representing a chromosome in circular form). The 10 rices are arranged in order of cultivated (1–3) and wild (4–10), and represented in evolutionary order from recently evolved to the oldest: 1-*Oryza sativa* var. *japonica*, 2-*Oryza sativa* var. *indica*, 3-*Oryza glaberrima*, 4-*Oryza rufipogon*, 5-*Oryza nivara*, 6*-Oryza barthii*, 7-*Oryza glumaepatula*, 8-*Oryza meridionalis*, 9-*Oryza punctate*, and 10-*Oryza brachyantha*. START types represented by circular glyphs/dots: blue, Minimal START; gray, SD; very light green, SM; yellow, PS; purple, PSD; red, HS; light orange, HZS; and green, HZSM.

The putative START coding genes were mapped on to chromosomes based on their gene location and karyotype information. Figure 2 depicts this for all 10 *Oryza* genomes and it is clear that despite variation in numbers, START genes show positional and structural conservation on the corresponding chromosomes, with slight variations in some genes reflecting syntenic block shuffling, which may in turn be due to (a) fragment rearrangements among chromosomes during speciation events, (b) isolated gene relocation events due to the homologs recombination or viral or transposon-based gene relocation mechanisms.

The START genes are distributed among 11 chromosomes (out of 12) across all wild and cultivated rices species (Figure 2), with the highest numbers mapping to chromosomes 8 and 10, while Chr 5 is devoid of any START genes (with a single exception of one START gene in early evolved *Omer*_*w*_). The HZSM class of START genes is predominantly located on chromosomes 1, 3, 10, and 12. Surprisingly, there are two HZS gene orthologs unequivocally present, one each on chromosomes 2 and 8 except in *Oniv*_*w*_ where one HZS gene was seen on Chr 10 instead of Chr 8. It may be recalled that SM, a special class of START that was not seen in any of the wild rices, occurs on Chr 1 amongst cultivated rices *Ojap*_*c*_ and *Ogla*_*c*_ and shares homology with HZSM on the same chromosome in eight other rices, suggesting a possible loss of HZ fragment of some members of the HZSM class. In contrast, the other SM gene on Chr 6 of *Ojap*_*c*_ showed homology with minimal START on Chr 6 of *Oruf*_*w*_, *Oniv*_*w*_, *Obar*_*w*_, and *Oglu*_*w*_ and is possibly an example of gain of function. These observations match the pairwise GSA patterns observed in the previous section and are further supported by the corresponding pairs of genes being orthologous as shown in Table 3. This table lists the orthologous genes in all 10 rices, using the recently evolved cultivated variety *Ojap*_*c*_ as reference for the other nine rice genomes, as described in section “Materials and Methods.” Interestingly, this table also shows that PS, (a special class of STARTs, seen only in the oldest wild rice *Obra*_*w*_) is orthologous to a member of the PSD class in the cultivated varieties. In contrast, the members of the SD class, observed only in cultivated *Oryza* species, and the oldest wild rice, are similar to each other but do not have any orthologs in other genomes of rice, not even in the immediate ancestors of the three cultivated varieties. Overall, the findings of this section support the idea of specialized functional roles for each of the eight START classes in plants, and we further explore this aspect in later sections.

**TABLE 3:** START genes in *Oryza sativa* var. *japonica* and their best orthologs amongst other nine rice species.

Cultivated rice species			Wild rice species						
*Oryza sativa* var. *japonica*	*Oryza sativa* var. *indica*	*Oryza glaberrima*	*Oryza rufipogon*	*Oryza nivara*	*Oryza barthii*	*Oryza glumipatula*	*Oryza meridionalis*	*Oryza punctata*	*Oryza brachyantha*
LOC_Os03g 01890_HZSM	BGIOSGA01 1687_HZSM	ORGLA03G0 005300_HZSM	ORUFI03G 00510_HZSM	ONIVA11G211 80_HZSM	OBART03G 00720_HZSM	OGLUM03G0 0670_HZSM	OMERI03G0 0480_HZSM	OPUNC03G 00630_HZSM OPUNC0 3G00670_HZSM	OB03G1 0760_HZSM
LOC_Os03g 43930_HZSM	BGIOSGA 013211_HZSM	ORGLA03G0 253300_HZSM	ORUFI03 G28280_HZSM	ONIVA03G 28370_HZSM	OBART03G 27200_HZSM	OGLUM03G 27880_HZSM	OMERI03G 00550_HZSM	OPUNC03G 24840_HZSM	OB03G35 020_HZSM
LOC_Os10g 33960_HZSM	BGIOSGA 033144_HZSM	ORGLA10G0 115700_HZSM	ORUFI10G 14170_HZSM	ONIVA10G14 800_HZSM	OBART10G 13430_HZSM	OGLUM10G 13240_HZSM	OMERI10G 10230_HZSM	OPUNC10G 11850_HZSM	OB10G2 0600_HZSM
LOC_Os12g4 1860_HZSM	BGIOSGA0 35845_HZSM	ORGLA12G0 158900_HZSM	ORUFI1 2G20830_HZSM	ONIVA12G 17580_HZSM	OBART12G 18630_HZSM	OGLUM12G 20280_HZSM	OMERI12G 14040_HZSM	OPUNC12G1 6840_HZSM	OB12G 25330_HZSM
LOC_Os01g 10320_SM	BGIOSGA 002186_HZSM	ORGLA01G 0060700_SM	ORUFI01 G06940_HZSM	ONIVA0 1G07970_HZSM	OBART01G 06400_HZSM	OGLUM01G 07380_HZSM	OMERI01G0 6500_HZSM	OPUNC01G0 6180_HZSM	OB01G1 6410_HZSM
LOC_Os06g 50510_SM			ORUFI06G 29670_mS	ONIVA06G 30280_mS	OBART06G 27690_mS	OGLUM06G 29150_mS			
LOC_Os02g4 5250_HZS	BGIOSGA0 05852_HZS	ORGLA02G 0238300_HZS	ORUFI02G 29080_HZS	ONIVA0 2G30520_HZS	OBART02G 27630_HZS	OGLUM0 2G28170_HZS	OMERI02G2 6880_HZS	OPUNC02G 25360_HZS	OB02G3 5000_HZS
LOC_Os08g1 9590_HZS	BGIOSGA02 8396_HZS	ORGLA08G 0080600_HZS	ORUFI08G 10670_HZS	ONIVA10G 10170_HZS	OBART08G 09450_HZS	OGLUM08G 10180_HZS	OMERI08G 08060_HZS	OPUNC08G 08700_HZS	OB08G 18460_HZS
LOC_Os01g 55549_HS	BGIOSGA 000782_HS	ORGLA01G0 257700_HS	ORUFI0 1G34950_HS	ONIVA01G 36080_HS	OBART0 1G31750_HS	OGLUM01G 35890_HS	OMERI01G 28810_HS	OPUNC01G 30780_HS	OB01G 40650_HS
LOC_Os01g 57890_HS	BGIOSGA 004594_mS BGIOSGA 004593_HS	ORGLA01G 0275300_HS	ORUFI01G 36780_HS	ONIVA01G 38360_HS	OBART01G 33600_HS	OGLUM01G 37810_HS	OMERI01G 30450_HS	OPUNC01 G32670_HS	OB01G42420_HS
LOC_Os04g 48070_HS	BGIOSG A014527_HS	ORGLA04G0 191300_HS	ORUFI04G 23720_HS	ONIVA04G 20660_HS	OBART04G 22070_HS	OGLUM04G 22100_HS	OMERI04G 18610_HS	OPUNC04G 19830_HS	OB04G29090_HS
LOC_Os04g 53540_HS	BGIOSGA 014304_HS	ORGLA04G0 231200_HS	ORUFI04G 27800_HS	ONIVA04G 25060_HS	OBART04G 26630_HS	OGLUM04G 26010_HS	OMERI04G 21680_mS	OPUNC04G 23630_HS	OB04G 32930_HS OB08G1 2470_HS
LOC_Os06g1 0600_HS	BGIOSGA0 21700_HS	ORGLA06G 0063600_HS ORGLA06G0 247800_HS	ORUFI06G 07070_HS	ONIVA06G 08130_HS	OBART06G 06830_HS	OGLUM06G 07200_HS	OMERI06G 08000_HS	OPUNC06 G06470_HS	OB06G 16250_HS
LOC_Os08g 04190_HS	BGIOSGA 027698_HS	ORGLA08G 0016400_HS	ORUFI08G 02510_HS	ONIVA08G0 2450_HS		OGLUM08G 02240_HS	OMERI08G 02310_HS	OPUNC0 8G02090_HS	
LOC_Os08g 08820_HS	BGIOSGA 028102_HS	ORGLA08G0 042800_HS	ORUFI08G 05670_HS	ONIVA08G 05020_HS	OBART08G 05070_HS	OGLUM08G 05340_HS		OPUNC08G 04870_HS	OB08G 15100_mS
LOC_Os09g 35760_HS	BGIOSGA 029405_HS		ORUFI09G 18410_HS	ONIVA09G 18120_HS	OBART09G 17090_HS	OGLUM09G 17630_HS	OMERI09G 12650_HS	OPUNC09G 15430_HS	OB09G 23660_HS
LOC_Os10g 42490_HS	BGIOSGA 031343_HS	ORGLA10G 0146400_HS	ORUFI10 G20730_HS	ONIVA10G 22060_HS	OBART10G 19440_HS	OGLUM10G 19540_HS	OMERI10G 15290_HS	OPUNC10G 17890_HS OPUNC10G 17920_HS	OB10G 26620_HS
LOC_Os02g 01270_PSD	BGIOSGA0 07330_PSD BGIOSGA 022187_PSD	ORGLA02G 0002000_SD	ORUFI02G 00290_PSD	ONIVA02G 00280_PSD ONIVA06G 01480_PSD	OBART02G 00270_PSD	OGLUM02G 00270_PSD OGLUM06G0 1090_PSD	OMERI02G0 9330_PSD OMERI06G 01090_PSD	OPUNC11G 05520_PSD	OB02G1 0290_PSD OB06G 11230_PS OB10G2 0570_PSD
LOC_Os10g 31770_PSD	BGIOSGA 033068_PSD	ORGLA10G 0106900_PSD	ORUFI10G 12680_PSD	ONIVA1 0G11380_PSD ONIVA10G 14690_PSD	OBART10G 12110_PSD	OGLUM10G 11870_PSD	OMERI10G 09100_PSD	OPUNC10G 10390_PSD	OB10G 19410_PSD
LOC_Os08g 34060_SD	BGIOSGA 028734_SD	ORGLA08G 0139700_SD							OB08G2 3520_SD
LOC_Os02g 03230_mS	BGIOSGA 007209_mS	ORGLA02G 0018400_mS	ORUFI02G 01930_mS	ONIVA02G 01830_mS	OBART02G 01950_mS	OGLUM02G 01810_mS	OMERI02G 02610_mS		
LOC_Os02g 26860_mS	BGIOSGA 008180_mS	ORGLA02G0 141000_mS	ORUFI02G1 6760_mS	ONIVA02G1 7540_mS	OBART02G 16280_mS	OGLUM02G 16340_mS	OMERI02G 15840_mS	OPUNC02G 14500_mS	OB02G24430_mS
LOC_Os04g 02910_mS	BGIOSGA 015687_mS	ORGLA04G 0008500_mS	ORUFI04G 01090_mS	ONIVA04G 00760_mS	OBART04G 01070_mS	OGLUM04G 00980_mS	OMERI04G 00950_mS	OPUNC04G 01070_mS	OB04G 10780_mS
LOC_Os06g 50560_mS									
LOC_Os06g 50724_mS	BGIOSGA 023612_mS								
LOC_Os07g0 8760_mS	BGIOSGA0 24704_mS	ORGLA07G0 045900_mS	ORUFI07G 05330_mS	ONIVA07G 04320_mS	OBART07G 05510_mS	OGLUM07G 04970_mS	OMERI05G 17970_mS	OPUNC07G 05340_mS	OB07G14050_mS
LOC_Os07g 47130_mS			ORUFI07G 26520_mS			OGLUM07G 25570_mS			
LOC_Os11g 14070_mS	BGIOSGA 034213_mS	ORGLA11G0 072100_mS	ORUFI11G 08470_mS	ONIVA11G 08330_mS	OBART11G 08150_mS			OPUNC11G 07930_mS	OB11G 16480_mS

### Phylogenetic Analysis of Different Structural Classes of START Domain Containing Proteins

**FIGURE 3:**
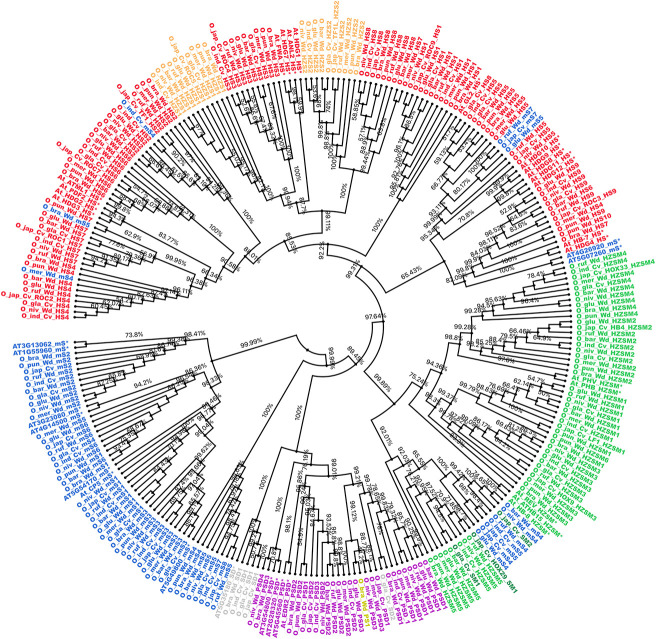
Cladogram for START proteins from all 10 *Oryza* genomes along with *Arabidopsis thaliana* (*). Color codes are same as earlier figures: red, HD START (HS); light orange, HD bZIP START (HZS); green, HD bZIP START MEKHLA (HZSM); dark green, START MEKHLA (SM); purple, PH START DUF1336 (PSD); yellow, PH START (PS); gray, START DUF1336 (SD); and blue for minimal START. Phylogeny codes for each locus ID are based on the orthologs analysis with reference to *Ojap*_*c*_ as described previously (see Supplementary Table 1).

The 249 START protein sequences from all 10 *Oryza* species and 35 reference sequences from the model plant *A. thaliana* were used to construct a phylogenetic dendrogram as described in section “Materials and Methods,” and this led to the grouping of genes having closely related evolutionary patterns as shown in Figure 3. The phylogenetic tree showed distinct clusters for all major structural classes of START domains, which suggests conservation amongst different structural classes of START proteins in terms of their sequences. Few of the minimal STARTs were distributed among different clusters that might be due to their vast differences in their sequence lengths. As shown in Figure 3, The HZSM represented in green forms a single distinct cluster, while HZS and HS represented in light orange and red, respectively, formed a single cluster, as expected with an overlap between these two subclasses. The two minor classes, i.e., PS (shown in olive) and SD (shown in gray) formed sub-cluster together with PSD (shown in purple). The minimal START proteins represented in blue forms a single large cluster, but some of them are distributed among other structural classes, which might be due to vast differences in their sequence lengths. The three SM class (represented in dark green) falls alongside HZSM and minimal START. As expected, all three cultivated rices were observed to lie adjacent to each other or in the same branch as their immediate wild ancestor.

Previous studies suggested that class III HD-ZIP proteins are evolutionarily conserved (Ariel et al., 2007). In this study, although this family formed a single cluster, few intervening minimal STARTs were also found. The unusual START type “START MEKHLA” also merged with this cluster, which shows evolutionary relatedness with class III HD-ZIP proteins, despite the lack of HD-ZIP region. The HD bZIP START (HZS) and HD START (HS) shared the high similarity between the two and which causes overlapping of clusters. Similarly, PS, PSD, and SD formed a single cluster.

### STARTS in Collinear Blocks – A Spatial Pattern Conservation of START Genes Among 10 *Oryza* Species

**FIGURE 4:**
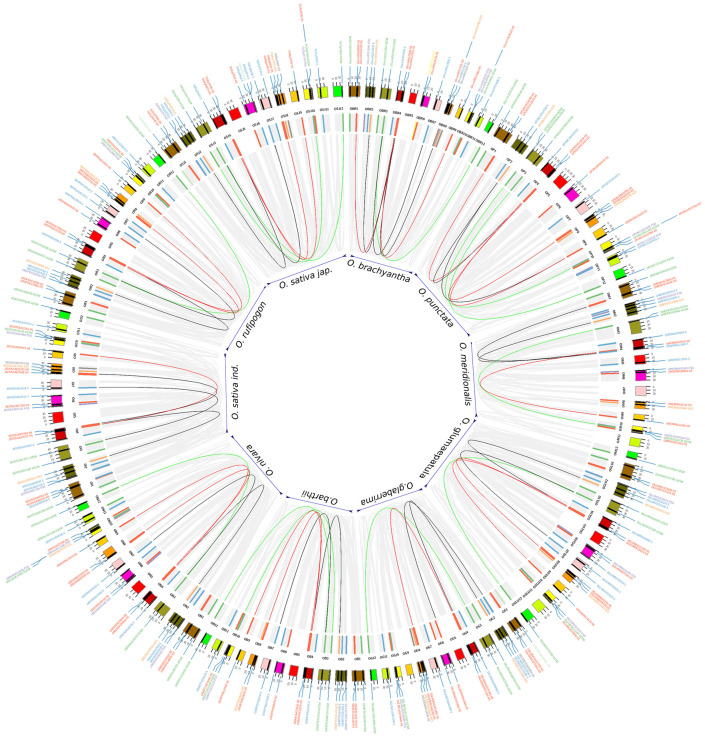
Self-collinear blocks of 10 *Oryza* species. Outer circle: 12 chromosomes from each of the 10 *Oryza* genomes that are represented in default colors of circos. Inner circle: START genes are shown as colored bar highlights. Color codes for bar highlights and labels are same as Figures 2, 3. Connectors (internal to the bar highlights) are formed between the START homologs that occur as collinear blocks on different chromosomes of the same genome and follows same color codes of START types except for START homologs that belongs to two different structural classes are linked with a black line. Gray connectors are used for showing non-START homologs within the genomes of 10 rices.

The occurrence of several genes into a collinear block provides clues on the spatial conservation of the individual genes and their proximal neighborhoods that provides the biological significance of gene blocks in the evolutionary sense. Figure 4 depicts these blocks as maps with START genes within one block linked by domain structural classes and collinear gene sets vary from 12 to 20% across the genome, the majority being close to 15% (Supplementary Table 3).

Six to ten START genes occur in collinear blocks, with about one-third gene number increase in the cultivated varieties, i.e., *Ojap*_*c*_ and *Oind*_*c*_ as compared to early evolved rice species *Obra*_*w*_, *Opun*_*w*_, or *Omer*_*w*_ and similar is the case of total gene numbers between AA genotype rice varieties *Ojap*_*c*_ (recently evolved) and *Omer*_*w*_ (early evolved). Lack of consistency in the number of genes in syntenic blocks hints at the possible chromosomal rearrangement of fragments bearing START genes in both wild and cultivated rice species. The patterns of collinear blocks of cultivated varieties *Ojap*_*c*_ appear similar to immediate ancestors *Oruf*_*w*_, unlike *Oind*_*c*_, each pair being placed next to each other in Figure 4. In contrast, the number of START genes in cultivated varieties is reminiscent of their wilder early evolved relatives, for instance, cultivated variety *Ojap*_*c*_ has 10 START genes in collinear blocks, like its indirect/wilder ancestors *Obar*_*w*_ and *Opun*_*w*_. The African domesticated varieties *Ogla*_*c*_, has seven START genes in its collinear blocks, equivalent to its wilder relative *Omer*_*w*_. Supplementary Table 3 provides a species-wise total number of collinear blocks and START genes that occur in these blocks, while individual circos maps of syntenic collinear blocks are provided in Supplementary Figures 2A–J.

### Identification of Different Modes of START Gene Duplication

**FIGURE 5:**
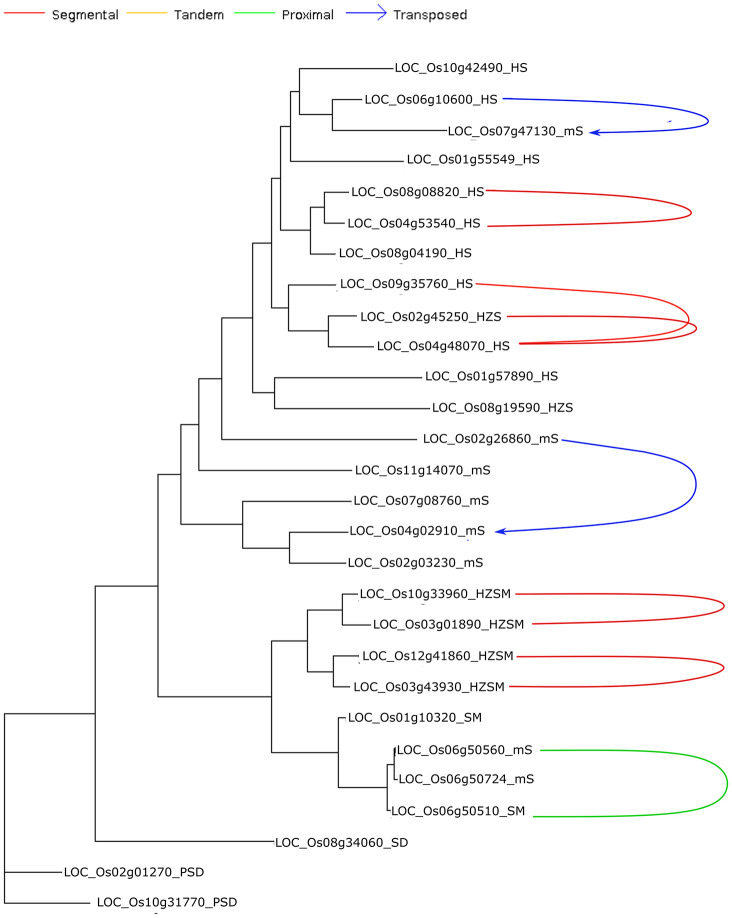
Phylogenetic tree of the *Oryza sativa* var. *japonica* START genes family annotated with different modes of gene duplication.

In plants, whole-genome duplication leading to polyploids is a frequent event, gene duplication being an important evolutionary phenomenon that helps in the gene dosage, adaptation and speciation; common modes being segmental duplication (SD), dispersed duplication (DD), tandem duplication (TD), and transposed duplication (TsD). Different modes of gene duplication were analyzed for START genes across 10 cultivated and wild *Oryza* species and revealed START genes to exist as duplicated pairs as shown in Table 4. As can be seen in Table 4, START genes are rarely present as singletons; and there are two major modes of gene duplication, namely, dispersed and segmental (arising from WGD) across the 10 species. Interestingly, dispersed and segmental duplications are similar between pairs of cultivated rice species and their immediate ancestors (*Ojap*_*c*_ and *Oind*_*c*_ with *Oruf*_*w*_; *Ogla*_*c*_ with *Obar*_*w*_) but the proximal and tandem START genes appear to have duplicated after speciation, as the immediate ancestors do not have any. Proximal and tandem duplicate modes among START genes are observed in only two of the early evolved wild species (*Omer*_*w*_ and *Opun*_*w*_). Figure 5 shows a START gene dendrogram with various modes of duplication and paralogous pairs for the main cultivated variety *Ojap*_*c*_, and it can be seen that of the eight pairs of duplicates, five pairs are segmentally duplicated (between chromosomes 2, 3, 4, 8, 9, 10, and 12), while the three START genes (all on Chr 6) are proximally duplicated. Besides these, two additional STARTS that are found in newly transposed locations as compared to their ancestral gene locations (Figure 5).

**TABLE 4:** Distribution of different modes of gene duplication based on whole genome and START genes (in parentheses) amongst 10 cultivated and wild *Oryza* species.

Name of plants	Singleton	Dispersed	Proximal	Tandem	WGD or segmental	Total
**Cultivated rice species**						
*Oryza sativa* var. *japonica*	8989 (1)	19,640 (15)	3266 (3)	4035 (0)	6259 (9)	42,189 (28)
*Oryza sativa* var. *indica*	7745 (1)	18,597 (17)	4599 (4)	5230 (1)	5860 (4)	42,031 (27)
*Oryza glaberrima*	7109 (1)	15,808 (16)	1783 (0)	3269 (0)	6161 (7)	34,130 (24)
**Wild rice species**						
*Oryza rufipogon*	9343 (1)	17,102 (17)	2741 (0)	2912 (0)	5814 (7)	37,912 (25)
*Oryza nivara*	9061 (1)	18,192 (18)	2605 (0)	2887 (0)	4281 (7)	37,026 (26)
*Oryza barthii*	8736 (1)	15,613 (13)	2749 (0)	3124 (0)	5331 (9)	35,553 (23)
*Oryza glumaepatula*	8916 (0)	16,191 (18)	2671 (0)	2971 (0)	5630 (7)	36,379 (25)
*Oryza meridionalis*	7667 (0)	14,172 (14)	2105 (1)	2567 (0)	3730 (7)	30,241 (22)
*Oryza punctata*	6721 (1)	14,027 (10)	2539 (3)	3245 (0)	6018 (10)	32,550 (24)
*Oryza brachyantha*	9330 (1)	13,561 (17)	1597 (0)	2690 (0)	5285 (7)	32,463 (25)

*(#) STARTs that occur in singleton, dispersed, proximal, tandem, and segmental duplication modes are mentioned in parentheses.*

### Nucleotide Substitution Rates and Ka/Ks Ratios

**FIGURE 6:**
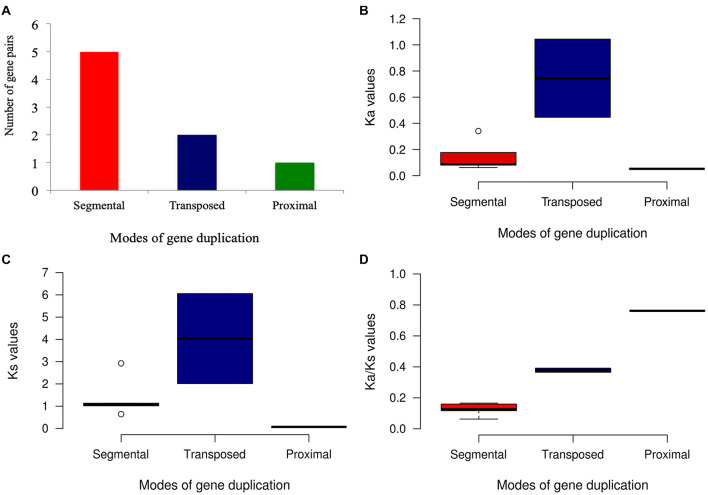
Evolutionary patterns of duplicated START gene pairs by different modes in *Oryza sativa* var. *japonica.* **(A)** Gene pair distribution among different modes of duplication. **(B)** Ka values of duplicated gene pair. **(C)** Ks value of duplicated gene pairs. **(D)** Ka/Ks values of duplicate genes pairs.

Ka/Ks ratios represent selection pressure on genes, with values of >1, <1, or 1 signifying positive, negative or neutral selection, respectively (Koonin and Rogozin, 2003). These calculations for START genes of all nine *Oryza* genomes with respect to recently evolved cultivated variety *Ojap*_*c*_ as described in section “Materials and Methods” are shown in Figures 6A–D and Supplementary Figures 3A–C. With few exceptions, most of the START gene pairs have Ka/Ks values below one, suggesting their being under negative selection. The unique domain categorical group of “SM” in *Ojap*_*c*_ and its orthologous pairs in *Oind*_*c*_, *Ogla*_*c*_, and *Oniv*_*w*_ showed a very high positive selection suggesting their being under positive selection. Apart from this, there are few other cases, which also showed Ka/Ks values significantly more than 1, and close to 1, which signifies that these START genes are also undergoing through positive selection. The analysis further confirmed a high rate of synonymous and non-synonymous substitutions for both the PSD type orthologs and single HS homolog (present on Chr 4).

A recent study showed that 99% of genes derived *via* duplication are under negative or purifying selection in rice (Qiao et al., 2019). In contrast, only 0.5% (WGD), 1.2% (tandem), 1.5 (proximal), 0.2 (transposed), and 1.4 (dispersed) gene pairs showed the positive selection pressure (Qiao et al., 2019). Estimation of synonymous (Ks) and non-synonymous (Ka) nucleotide substitution rates gave an important insight on evolution of duplicated gene pairs. The higher synonymous mutation rate (Ks) indicated the long evolutionary history of the respective genes in their genomes, thus, highlighting the functional importance for the retention of the gene copies (Ren et al., 2014; Qiao et al., 2018). The eight paralogous START gene pairs of *Ojap*_*c*_ that were noticed in segmental, transposed and proximal modes of duplications (Figure 5) were further evaluated for the Ka, Ks, and Ka/Ks. As shown in Figure 6, all gene pairs have negative Ka/Ks ratios suggesting low or moderate flexibility for mutational changes. One proximal duplicate pair (LOC_Os06g50560_mS-LOC_Os06g50510_SM) at 0.762 suggests flexibility in the mutational rate of the second copy gene, whereas, for the five segmental START gene duplicates, Ka/Ks varied between 0.166 and 0.063, indicating stringent regulation and highlighting their functional importance (LOC_Os10g33960_HZSM-LOC_Os03g01890_HZSM, LOC_Os 12g41860_HZSM-LOC_Os03g43930_HZSM, LOC_Os08g088 20_HS-LOC_Os04g53540_HS, LOC_Os04g48070_HS-LOC_Os 02g45250_HZS, and LOC_Os04g48070_HS-LOC_Os09g35 760_HS) (detailed analysis provided in Supplementary Table 4). As presented in the next section, we also observed similar expression levels among these pairs across anatomical parts but with slight variation between different stages of development.

Overall, Ka/Ks analysis of duplicated START gene pairs in *Ojap*_*c*_ suggest that the expanded START gene family is still evolving toward stabilization of function and expanding into new roles or sub-functionalization. The Ka/Ks results also suggest that proximally duplicated STARTs have 99% of identity amongst themselves and have not undergone changes, compared to other modes of duplications, which might be due to the recent incidence of this mode of duplication. Further, segmentally duplicated START gene pairs showed low Ka values and Ka/Ks ratio, alongside of having relatively high Ks values, suggesting evolution under stringent selection pressures over a long time. This is also supported by the phenomenon of the overall number of accumulated mutations over the evolutionary history of an organism (Zhu et al., 2014). Contrastingly, the transposed duplicates underwent intermediate negative selection pressure, and the segmental duplicates underwent strong negative selection pressures. Similarly, the synonymous substitution rates for transposed duplicates were higher when compared to segmental START duplicates. The transposed pair LOC_Os02g26860_mS-LOC_Os04g02910_mS showed threefold higher Ka and Ks values supporting the phenomenon of evolutionary freeness for development of sub-functionalization or neo-functionalization when compared to segmental duplicate gene pair, i.e., LOC_Os04g48070_HS-LOC_Os09g35760_HS, which showed similar Ka and Ks values with other transposed pair LOC_Os06g10600_HS-LOC_Os07g47130_mS showing slight stringency in mutational frequency of these genes.

The transposed pairs (LOC_Os02g26860_mS-LOC_Os04 g02910_mS and LOC_Os06g10600_HS-LOC_Os07g47130_mS; above 0.365 Ka/Ks ratio) in addition to the proximal duplicated pairs (LOC_Os06g50560_mS-LOC_Os06g50510_SM; 0.762 Ka/Ks ratio) had the highest mean Ka/Ks ratio indicating that they have experienced weaker purifying selection. The segmental gene pair (LOC_Os10g33960_HZSM-LOC_Os03g0 1890_HZSM) had the lowest mean Ka/Ks ratio (0.063) suggesting strong purifying selection and the other four segmental pairs (LOC_Os12g41860_HZSM-LOC_Os03g43930_HZSM, LOC_Os08g08820_HS-LOC_Os04g53540_HS, LOC_Os04g4 8070_HS-LOC_Os02g45250_HZS, and LOC_Os04g48070_HS-LOC_Os09g35760_HS) with intermediary mean Ka/Ks ratio above 0.1, indicating that they had experienced intermediate to stronger purifying selection. Thus, START genes appear to be under purifying selection pressure, further highlighting their functional importance and roles for expansion of START genes among wild and cultivated rices, and we explore this further through gene expression analyses.

### Transcriptome Analysis of START Encoding Genes

**FIGURE 7:**
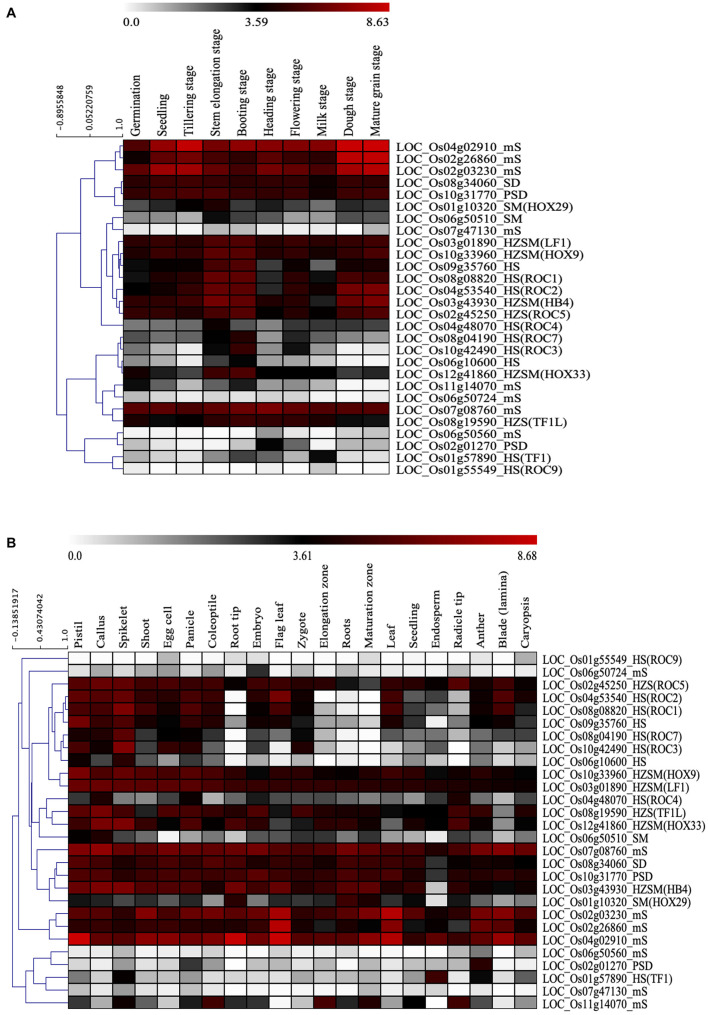
*In silico* expression study of 28 START genes in *Oryza sativa* var. *japonica*. **(A)** Hierarchical gene expression pattern of START genes at different developmental stages. **(B)** Hierarchical gene expression pattern of START genes in various anatomical parts.

The function of many START genes especially HD associated START genes have been extensively studied in plants. The class III HD-ZIP family and class IV HD-ZIP family have well-established roles in *Arabidopsis* and involved in various stages of development and gene regulation (Ariel et al., 2007). In order to explore the potential functions of the 28 START genes found in *O. sativa* var. *japonica*, the tissue and developmental stage-specific expression patterns were investigated in non-stressed condition as described in section “Materials and Methods.” As can be seen from Figures 7A,B and Supplementary Figures 4A,B, the expression heat maps of START genes shows, four HZSM, two HD bZIP STARTs, and two START MEKHLA genes in *O. sativa* var. *japonica* showed significant expression throughout the developmental stages and anatomical parts. Further, five out of nine HD-START genes express constitutively through the various developmental stages, but almost all nine genes showed differential expression across various anatomical parts, suggesting tissue specific roles for this largely amplified sub-group. The eight minimal START genes (LOC_Os02g03230_mS, LOC_Os02g26860_mS, LOC_Os04g02910_mS, LOC_Os06g50560_mS, LOC_Os06g 50724_mS, LOC_Os07g08760_mS, LOC_Os07g47130_mS, and LOC_Os11g14070_mS) of *Ojap*_*c*_ showed a wide variation in expression patterns across all stages and tissues, and it is possible to assign them to tissue-specific roles, with only one (LOC_Os07g08760_mS) showing high expression across all anatomical parts. START genes were grouped *via* hierarchical clustering of expression profiles and this is depicted in Figures 7A,B. Comparison of this data with duplication analyses in the earlier section reveals that most of the duplicated START gene pairs showed similar expression pattern across both developmental stages and anatomical parts, except for proximal duplicated START genes (detailed analysis provided in Supplementary Table 4). Expression patterns among five segmental gene pairs varied with three pairs in one cluster and two in other clusters, despite showing significant expression throughout all the developmental stages. The five segmental pairs constitute two pairs of HS genes (four genes) and they cluster together in expression as well. Taken together (Figures 5, 7) duplicated START genes in segmental and transposed modes showed a unified pattern in gene expression amongst duplicated gene pairs across all the developmental stages as well as in all anatomical parts, which signifies the functional importance of STARTs and the necessity of retaining both the copies of the gene pairs. Contrastingly, the proximal duplicate gene pair showed an uneven expression pattern between the gene pairs, indicating sub-functionalization or neo-functionalization of the duplicated genes, as was observed for the Ka/Ks selection pressures.

## Discussion

Genome duplication events play a significant role in environmental adaptation and speciation of organisms. Study on post duplication events has shed light on genome evolution and functional diversification, while studies on loss of alternate copies of duplicated loci have shown species divergence (Mizuta et al., 2010). Major changes upon post-genome duplication events, are copy gain or loss (that alters dosage), and domain alterations (e.g., gain, loss, or rearrangements) that regulate environmental adaptation (Kassahn et al., 2009; Yang and Bourne, 2009; Panchy et al., 2016; Qiu et al., 2017). Despite availability of 23 wild and cultivated rice accessions representing 11 genome types (AA, BB, CC, EE, FF, GG, BBCC, CCDD, HHJJ, HHKK, and KKLL), most previous studies have been limited to phylogenetic inferences, that too for gene families involved in transcriptional activation or repression of mainstream physiological processes (Ma and Bennetzen, 2004; Ammiraju et al., 2008; Jacquemin et al., 2014; Zhang et al., 2014; Zhong et al., 2019). In the current study, we have attempted to explore the START gene family across seven wild and three cultivated rice varieties to understand evolutionary changes related to copy number variation (CNV), alteration in mutational rates due to selection pressures, and combined these with present day functional divergence based on gene expression and domain conservation. Despite several studies on HD associated START proteins in plants (Schrick et al., 2004, 2014; Mukherjee and Bürglin, 2006; Chew et al., 2013; Pandey et al., 2016), present study is the first comprehensive comparison of this family between wild and cultivated rices.

### Gene Family Expansion and Copy Number Variation of START Genes in Rice

The START genes are well known to be amplified in plants compared to animals, but their presence in evolutionarily distant kingdoms of bacteria as well as protists, has led to questions about mechanism of amplification during family evolution, and how this amplification may have affected functional diversity of this group in present day plants (Iyer et al., 2001; Schrick et al., 2004). Newer versions of genome assemblies have enabled us to update these numbers and we find an increase in the number of START genes in *Ojap*_*c*_ (based on MSU Release 7.0).

Gene dosage plays a significant role in the metabolic and phenotype changes, which in turn decides species’ adaptation to the environmental changes (abiotic stress) and biotic stress in many plant families. CNVs was observed to change from segmental duplicates into both dispersed and proximal START models between the evolutionarily related rice varieties, i.e., *Obra*_*w*_ and *Opun*_*w*_; *Ojap*_*c*_ and *Oind*_*c*_. However, START gene copies mostly occur as dispersed duplicates, which additionally determines the total number of START genes in different *Oryza* genomes (Table 4). Evolution of the rices has seen several genotype changes from FF (*Obra*_*w*_; wild variety) to AA (*Oind*_*c*_ and *Ojap*_*c*_; Asian cultivated varieties) in the diploid rices (Yu et al., 2005). Overall, our results are in concordance with the rice genome evolution from FF to AA genotype which affected the START gene homolog location change, and loss of a copy in some genotypes like HS, PSD, and minimal START (Tables 1, 3 and Supplementary Table 3).

### Sub Genomic Distributions and Syntenic Relationships Among Rice START Genes

We observed a significant change in the positional change of START genes among different chromosomes between the BB and AA genomes, which may correlate well with the increase in the number of chromosomal inversions that were reported. Stein et al. (2018) observed that AA-genome-specific inversion was seen in *Omer*_*w*_ after the split with BB-genome (*Opun*_*w*_) where we have also observed a drop in the START gene numbers (Stein et al., 2018). Additionally, we have noted a shift in START gene numbers between the segmentally duplicated to dispersed START genes. A drastic change in the overall genome level collinear blocks was observed in the genomes of *Omer*_*w*_ and *Oniv*_*w*_, with the former showing loss of chromosomal fragments resulting in shorter genome size, which may be the root cause of fewer START gene numbers. Furthermore, our results showed proximal START duplicates in *Ojap*_*c*_ (AA), *Oind*_*c*_ (AA), and *Opun*_*w*_ (BB), indicating domestication as a cause of individual gene duplications in both *Ojap*_*c*_ and *Oind*_*c*_, but the proximal duplicates in *Opun*_*w*_ may be ascribed to the long evolutionary history between *Obra*_*w*_ and *Opun*_*w*_ (approximately 8.24 million years ago) (Stein et al., 2018). A special kind of START, i.e., SD-type was only seen in the *Obra*_*w*_ (earliest evolved), *Ogla*_*w*_, *Oind*_*c*_, and *Ojap*_*c*_ but absent in six other *Oryza* species. Several mS START homologs were found to be missing between different *Oryza* species indicating species level chromosomal rearrangements (Table 3 and Supplementary Figures 2A–J). Additionally, three proximal duplicates were identified on Chr 6 of *Ojap*_*c*_ but these genes belong to different types (two mS and one SM which showed similarity in exon–intron patterns for START domain encoding regions). Similar is the case for the single tandem START gene pair that was observed in the *Oind*_*c*_ on Chr1 (one HS type and one minimal mS).

Multiple reports support the idea of an initial polyploidization event in rice, followed by stabilization at the diploid level, after several rounds of genome rearrangements and gene loss (Wang et al., 2005; Yu et al., 2005; Stein et al., 2018). These patterns agree well with our observation that 95% of *Ojap*_*c*_ START genes are ancient duplicates. We found all possible modes of duplications, including WGD.

### Selection Pressure and Evolutionary Fates of Rice START Domains

The START domains were classified into distinct classes based on the presence of additional functional domains and their mutual arrangements/location on the sequence. The presence/absence of additional conserved domains can often provide insights into the divergence of a gene family, or the extent and direction of sub-functionalization among its members. The domain structural classes of START domains across 10 rice genomes provided insights into the distribution of each group among START proteins, as well as in subsequent comparative analyses, such as gene structure, evolutionary conservation, and expression patterns. By comparing these patterns across wild and cultivated rice genomes, we gained further insights into frequency of each domain structural class among closely related species, in terms of species divergence and functional significance of these structural classes. For the duplicates within each domain structural class, we further investigated gene family expansions, which revealed stringency in selection bias and Ka/Ks values below one indicating their functional importance in attaining the species adaptability and environmental robustness (Bokros et al., 2019). Wang et al. (2005) reported that 47% of the total genes in *O. sativa* var. *indica* genome were detected in 10 duplicated blocks among the 12 chromosomes, of which we have observed two START gene duplicate pairs among the eight pairs on the largest duplicated block region between Chr 2 and Chr 4, which in turn, was further shown to be a result of large-scale duplication events that occurred c. 70 million years ago, as inferred from phylogeny (Wang et al., 2005). Expression levels and domain structure changes suggest that change in bZip regions may signify loss in function for the HS class. Very large Ks and Ka values were recorded for the PSD STARTs, indicating long evolutionary history and functional importance. The lower stringency in Ka/Ks ratio for transposed and proximal START gene pairs indicates incomplete sub-functionalization or neo-functionalization states of these genes. Interestingly, the difference between domain structure of wild and cultivated rice was not observed among homeodomain containing STARTs, and this may reflect the importance of regulatory domains during evolution. HD associated STARTs in plants are crucial for development starting from germination to maturation. The higher number and uniformity of HD associated START can also be explained by the previous observations of Freeling (2009), which suggests that gene retention after duplication shows a biased trend toward those duplicated genes that play important roles in plant functioning and survival (Freeling, 2009). Another cause for this uniformity may be localization, which in turn is associated with the conserved synteny pattern during genome duplication.

### Transcriptome and Proteome Level Patterns Across Domain Structural Classes

We assessed evolutionary significance of novel START domain structural classes from their expression levels in terms of anatomy and development. PH and START domain containing proteins (PSD class) showed expression in the early seed germination phase as well as many floral and vegetative tissues, with loss-of-function mutant developing resistance to powdery mildew (Tang et al., 2005). There are very few reports on proteome level changes of post-genome duplications (Kersting et al., 2012; Finet et al., 2013), but these early reports, support our data on the formation of novel START classes such as PS and SM, arising from domain gain/loss. Our results also suggest the possibility of two independent truncation events that may have led to the formation of SM subclades of START proteins either from HZSM clade or mS clade. There may be a possible gain/loss in the PSD structural class leading to PS subclade or vice versa. Kersting et al. (2012) have report on the domain loss in monocots strengthens the idea of possible domain loss mechanism in certain classes of START proteins to form novel structural classes. Additionally, their data show higher domain gain/loss events in *O. sativa* genome than *Brachypodium distachyon*. These reports support the involvement of domain level changes in adaptability of plants to environmental changes. Stein et al. (2018) have shown a total of nine evolutionarily conserved HD-bZIP containing proteins in Oryza that originated at the Magnoliophyta taxon. We have shown the presence of six to eight HD-bZIP containing START proteins among those nine in various structural forms across all wild and cultivated rices investigated here. Genome divergence played a major role in this variation. Divergence of FF genome (*Obra*_*w*_) to BB genome (*Opun*_*w*_) showed an extra copy of the HZSM due to proximal duplication on Chr 3 in *Opun*_*w*_ which in the subsequent evolution to AA genome showed the loss of the extra HZSM gene copy that retained all the original numbers of *Obra*_*w*_ except two Oryza AA genomes, i.e., *Ogla*_*c*_ and *Ojap*_*c*_ but they showed a truncation of the HZ region leaving the SM type (Table 1). Although the *Ogla*_*c*_ and *Ojap*_*c*_ are evolutionarily far when compared to the *Ojap*_*c*_ and *Oind*_*c*_ our observation of the presence of the SM type homologs in an identical chromosomal location in the *Ogla*_*c*_ and *Ojap*_*c*_ contradicts the phylogenetic origination of the Oryza genomes. Apart from this, an additional copy of the SM type is seen on Chr 6 as a proximal duplicate in *Ojap*_*c*_. Expression divergence among duplicates that occurred through distinct modes of duplications has been reported earlier for *Oryza* and *Arabidopsis*, i.e., transposed duplicates > dispersed duplicates > proximal > WGD/segmental duplicates = tandem duplications, where the WGD and tandem duplicate pairs are more likely to maintain their original expression pattern (Wang et al., 2011). We find a very similar expression divergence among the different START duplicate pairs in rice genome. Overall, we find that gene gain and loss events have occurred at both individual genes as well as in collinear gene sets for the START genes among different cultivated *Oryza* genomes, which was evident from absence of homologous gene copies from their respective ancestral genomes.

In summary, we hope that the current comparative genomics analysis in wild and cultivated rice varieties will pave the way for experimental validation of these homologs in *Oryza*, a major food source for the world population. In addition the recent developments in the commercial-scale production of the rice bran oil highlights the importance of the future experimental studies in establishing the roles of START proteins in plants fatty acid metabolic pathway especially in commercial oilseed crops research. These novel domain combinations in addition to their huge gene CNVs in plants highlights their varied functional roles.

## Conclusion

The START domains are abundant in plants and play a crucial role in plant physiology and development. In this work, we have identified START family proteins in 10 wild and cultivated rice genomes and classified these into distinct structural classes based on functional domains. A detailed phylogenetic analysis was performed to map evolutionary divergence among these structural classes, followed by the superimposition of the data onto wild and cultivated rice varieties, revealing interesting features and patterns of evolution and ancestry of these domains within the 10 species investigated, which further helped us to understand START gene family expansion during domestication of rice. Most importantly, we find gene duplication/ontogeny to recapitulate selection pressures during domestication, revealing the indispensability and crucial roles performed by the START family. Patterns of gene duplication were superimposed on gene expression profiles for the most widely used rice variety across the globe, namely *O. sativa* var. *japonica*, further confirming functional aspects and divergence of this gene family in plant development and tissue specific roles. We hope this work on START gene family in *Oryza* species will pave the way for exploring the functional mechanism and substrate preference of plants START domains.

## Data Availability Statement

Publicly available datasets were analyzed in this study. This data can be found here: the complete genomic sequences, protein sequences, and annotation information of *Oryza brachyantha* (v1.4b), *Oryza punctata* (v1.2), *Oryza meridionalis* (v1.3), *Oryza glumaepatula* (v1.5), *Oryza barthii* (v1), *Oryza nivara* (v1.0), *Oryza rufipogon* (OR_W1943), *Oryza glaberrima* (v1), and *Oryza sativa* var. *indica* (ASM465v1), were downloaded from EnsemblPlants (http://plants.ensembl.org/info/data/ftp/index.html). The similar data for the main cultivated variety, *Oryza sativa* var. *japonica* (MSU Release 7.0) was downloaded from the Phytozome v12 (https://phytozome.jgi.doe.gov/pz/portal.html#!info?alias=Org_Osativa). All of the datasets supporting the results of this article are included within the article and its Supplementary Material.

## Author Contributions

GY conceived the work. SKM and RKP performed the research work. All authors performed the data analysis, wrote the manuscript, and approved for final publication.

## Conflict of Interest

The authors declare that the research was conducted in the absence of any commercial or financial relationships that could be construed as a potential conflict of interest.

## Publisher’s Note

All claims expressed in this article are solely those of the authors and do not necessarily represent those of their affiliated organizations, or those of the publisher, the editors and the reviewers. Any product that may be evaluated in this article, or claim that may be made by its manufacturer, is not guaranteed or endorsed by the publisher.

## Footnotes

1
http://hmmer.org/

2
http://tree.bio.ed.ac.uk/software/figtree/

## Supplementary Material

Supplementary Figure 1 Gene structure analysis of STARTs genes among 10 rice species **(A)** *Oryza sativa* var. *japonica*, **(B)** *Oryza sativa* var. *indica*, **(C)** *Oryza glaberrima*, **(D)** *Oryza rufipogon*, **(E)** *Oryza nivara*, **(F)** *Oryza barthii*, **(G)** *Oryza glumaepatula*, **(H)** *Oryza meridionalis*, **(I)** *Oryza punctata*, and **(J)** *Oryza brachyantha.*

Supplementary Figure 2 Collinear blocks for the 10 rice genomes **(A)** *Oryza sativa* var. *japonica*, **(B)** *Oryza sativa* var. *indica*, **(C)** *Oryza glaberrima*, **(D)** *Oryza rufipogon*, **(E)** *Oryza nivara*, **(F)** *Oryza barthii*, **(G)** *Oryza glumaepatula*, **(H)** *Oryza meridionalis*, **(I)** *Oryza punctata*, and **(J)** *Oryza brachyantha.*

Supplementary Figure 3 **(A)** Ka, **(B)** Ks, and **(C)** Ka/Ks values for START homologs of different *Oryza* species with respect to *Oryza sativa* var. *japonica.*

Supplementary Figure 4 Conditional gene expression pattern of START genes for **(A)** different developmental stages **(B)** various anatomical parts.

Supplementary Table 1 The locus ids of START genes along with sequence analysis information and phylogenetic code.

Supplementary Table 2 The detailed gene structure pattern of 10 *Oryza* genome, 5′-UTR, 3′-UTR, exon and Intron length.

Supplementary Table 3 Collinear genes number across 10 *Oryza* genome.

Supplementary Table 4 Ka, Ks, and Ka/Ks analysis among the gene pairs that follow different modes of duplication in *Oryza sativa* var. *japonica* genome.
