# Supplementary material for: StAR-Related Lipid Transfer (START) Domains Across the Rice Pangenome Reveal How Ontogeny Recapitulated Selection Pressures During Rice Domestication
Source: Front Genet. 2021 Sep 8;12:737194. doi: 10.3389/fgene.2021.737194 (PMC8455945; doi:10.3389/fgene.2021.737194)
Supplement: Supplementary Figure 1 — Gene structure analysis of STARTs genes among 10 rice species (A) Oryza sativa var. japonica, (B) Oryza sativa var. indica, (C) Oryza glaberrima, (D) Oryza rufipogon, (E) Oryza nivara, (F) Oryza barthii, (G) Oryza glumaepatula, (H) Oryza meridionalis, (I) Oryza punctata, and (J) Oryza brachyantha. [file Data_Sheet_1.PDF]

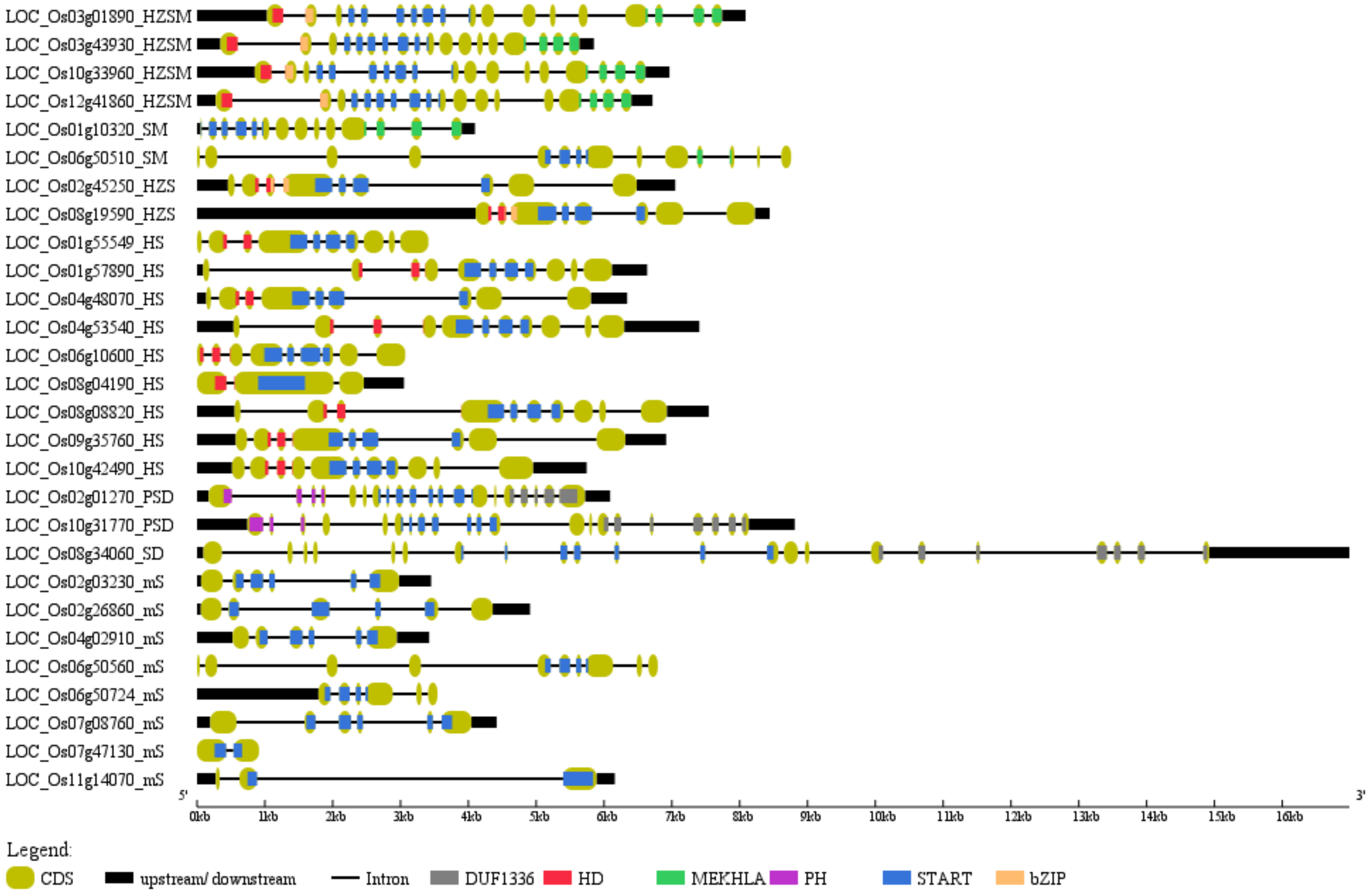

Supplementary Figure 1A. Gene structure analysis of the twenty-eight STARTs in *Oryza sativa* var. japonica: exon-intron patterns

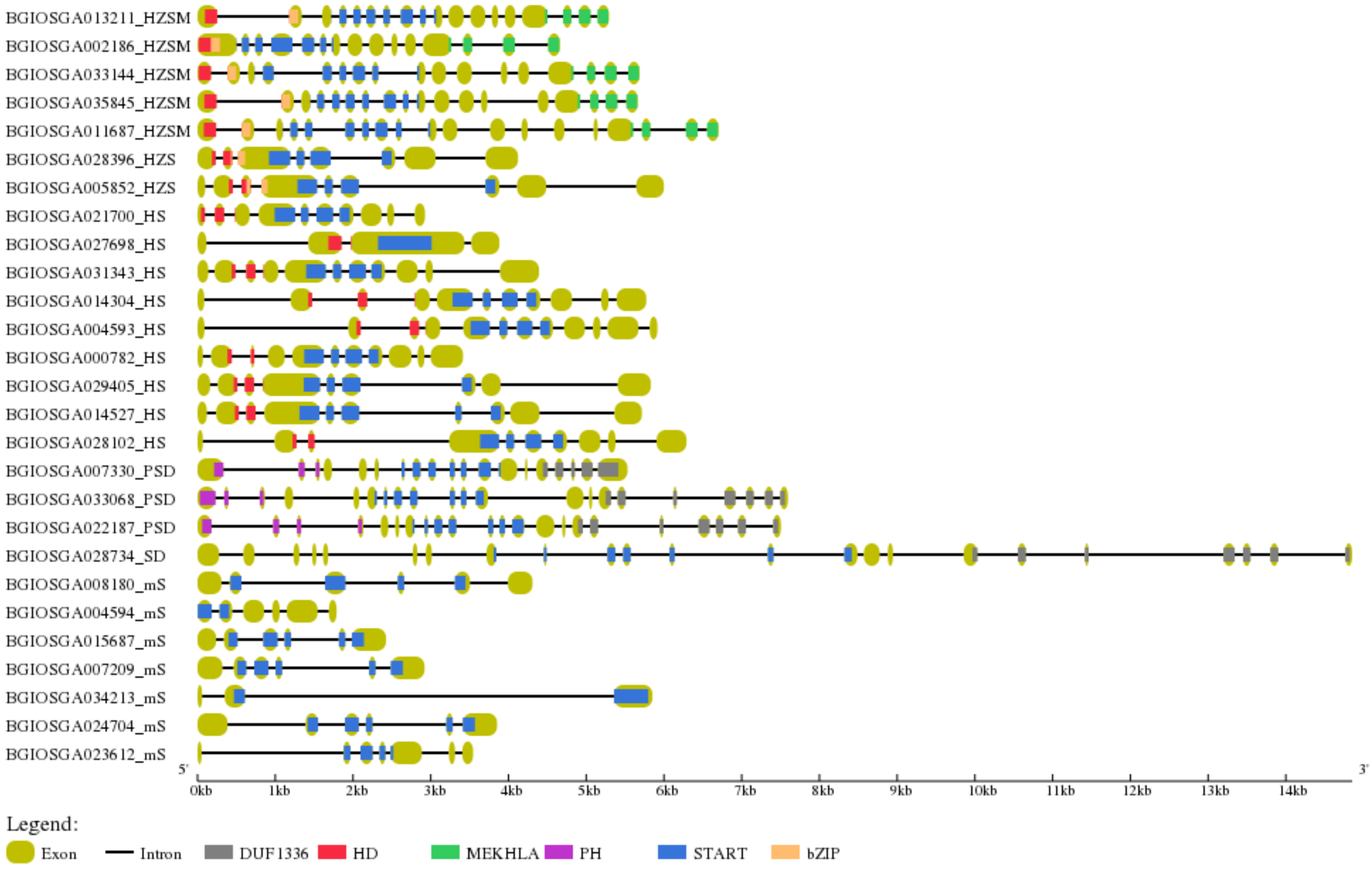

Supplementary Figure 1B. Gene structure analysis of the twenty-seven STARTs in *Oryza sativa var. indica*: exon-intron patterns

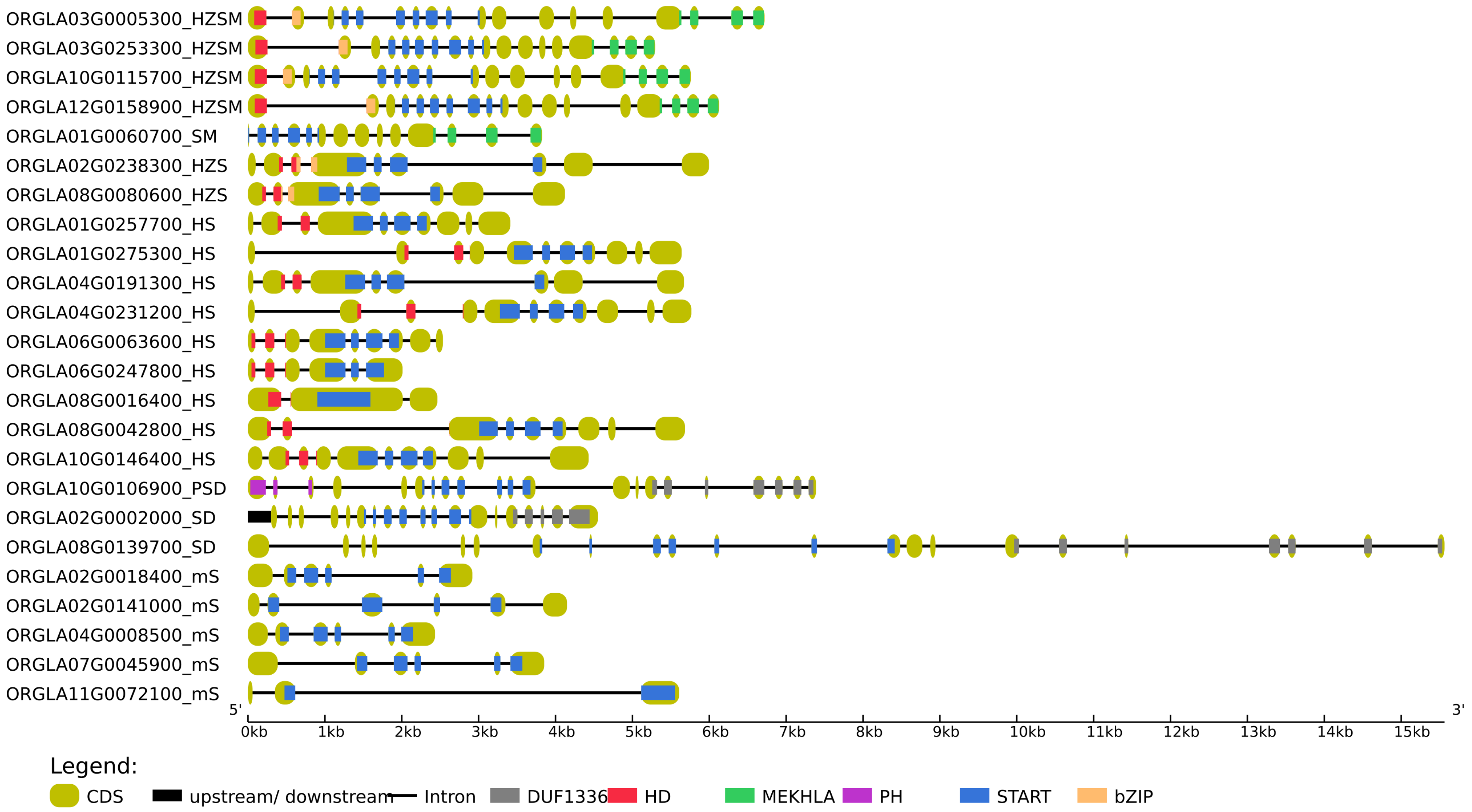

Supplementary Figure 1C. Gene structure analysis of the twenty-four STARTs in *Oryza glaberrima*: exon-intron patterns

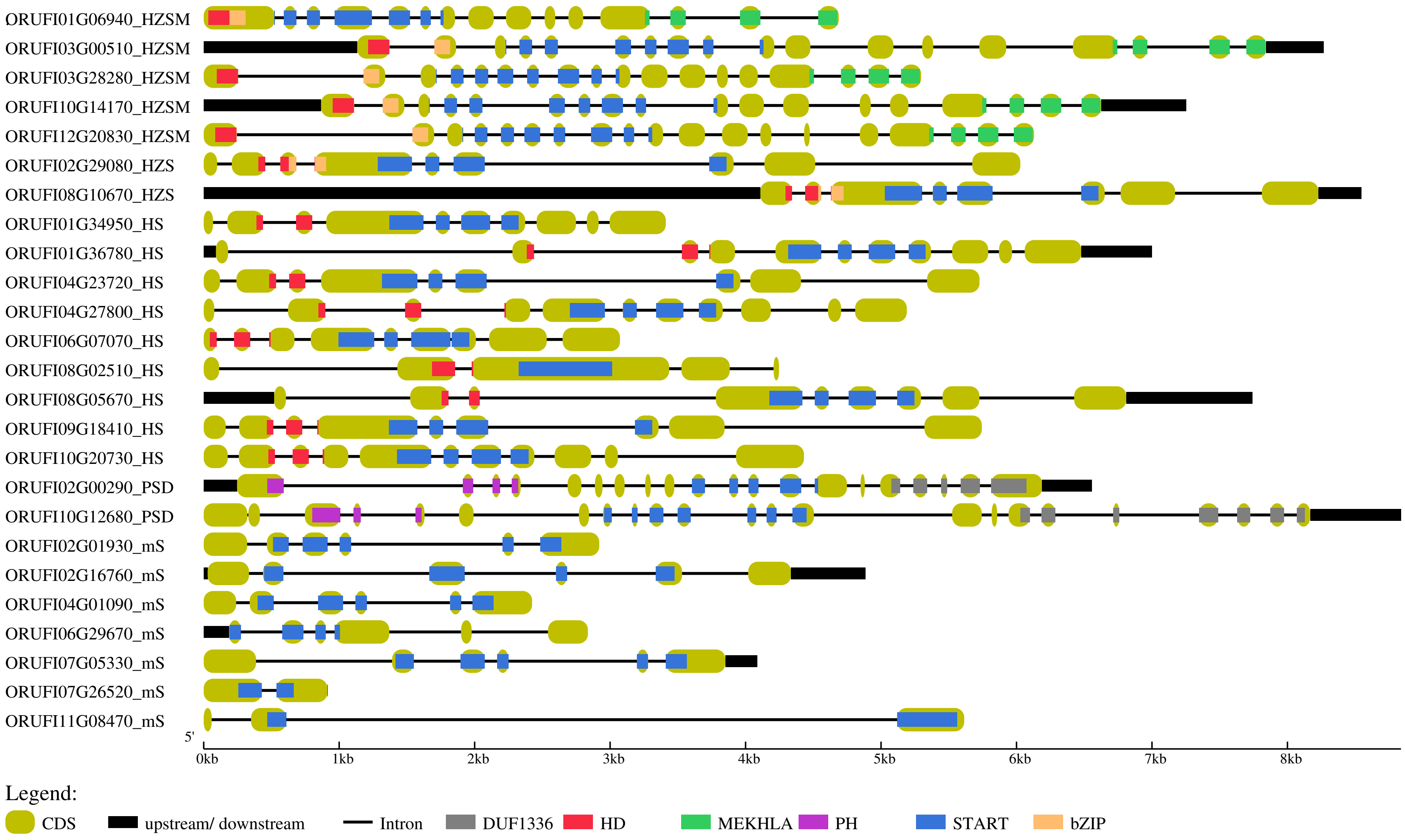

Supplementary Figure 1D. Gene structure analysis of the twenty-five STARTs in *Oryza rufipogon*: exon-intron patterns

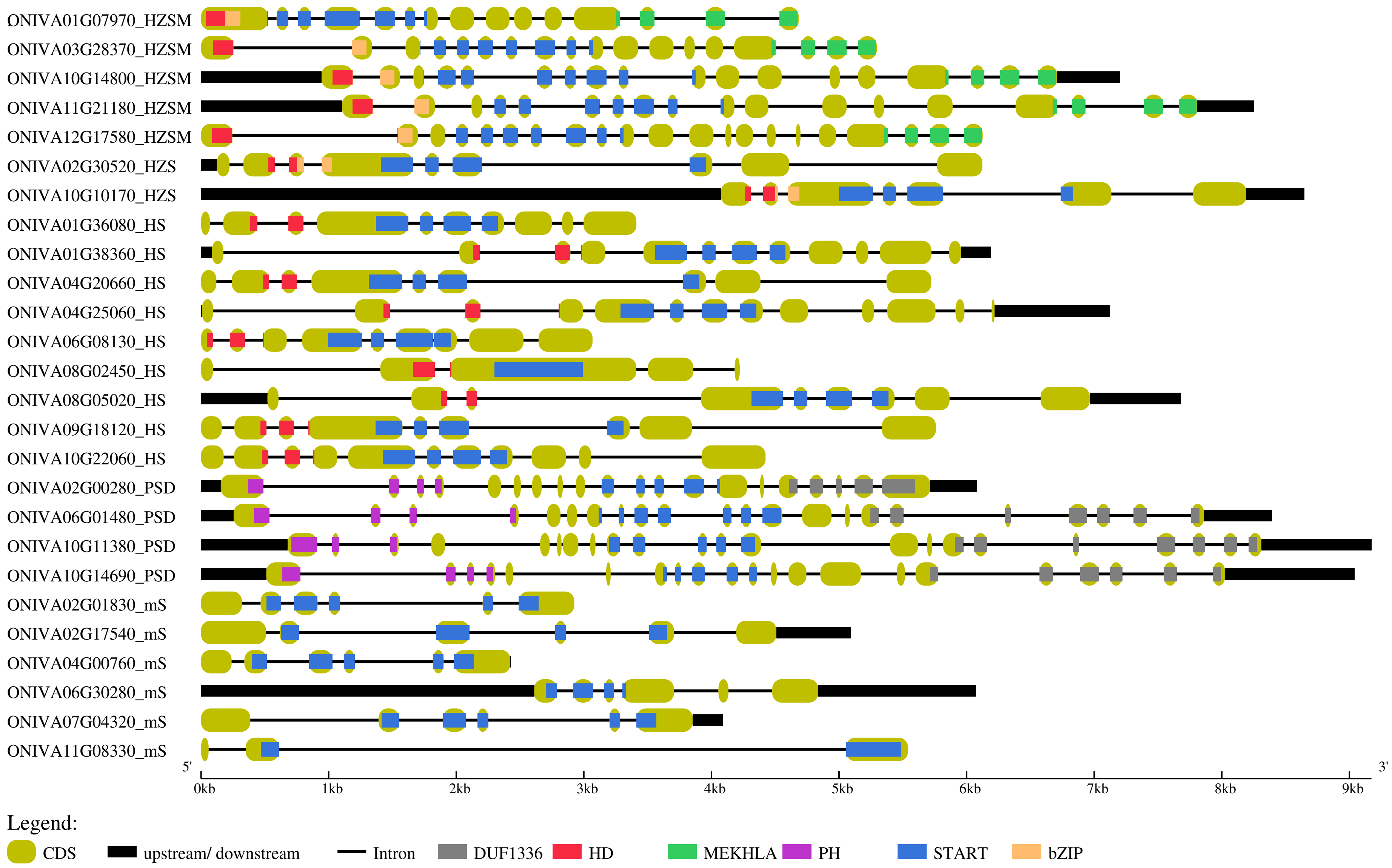

Supplementary Figure 1E. Gene structure analysis of the twenty-six STARTs in *Oryza nivara*: exon-intron patterns

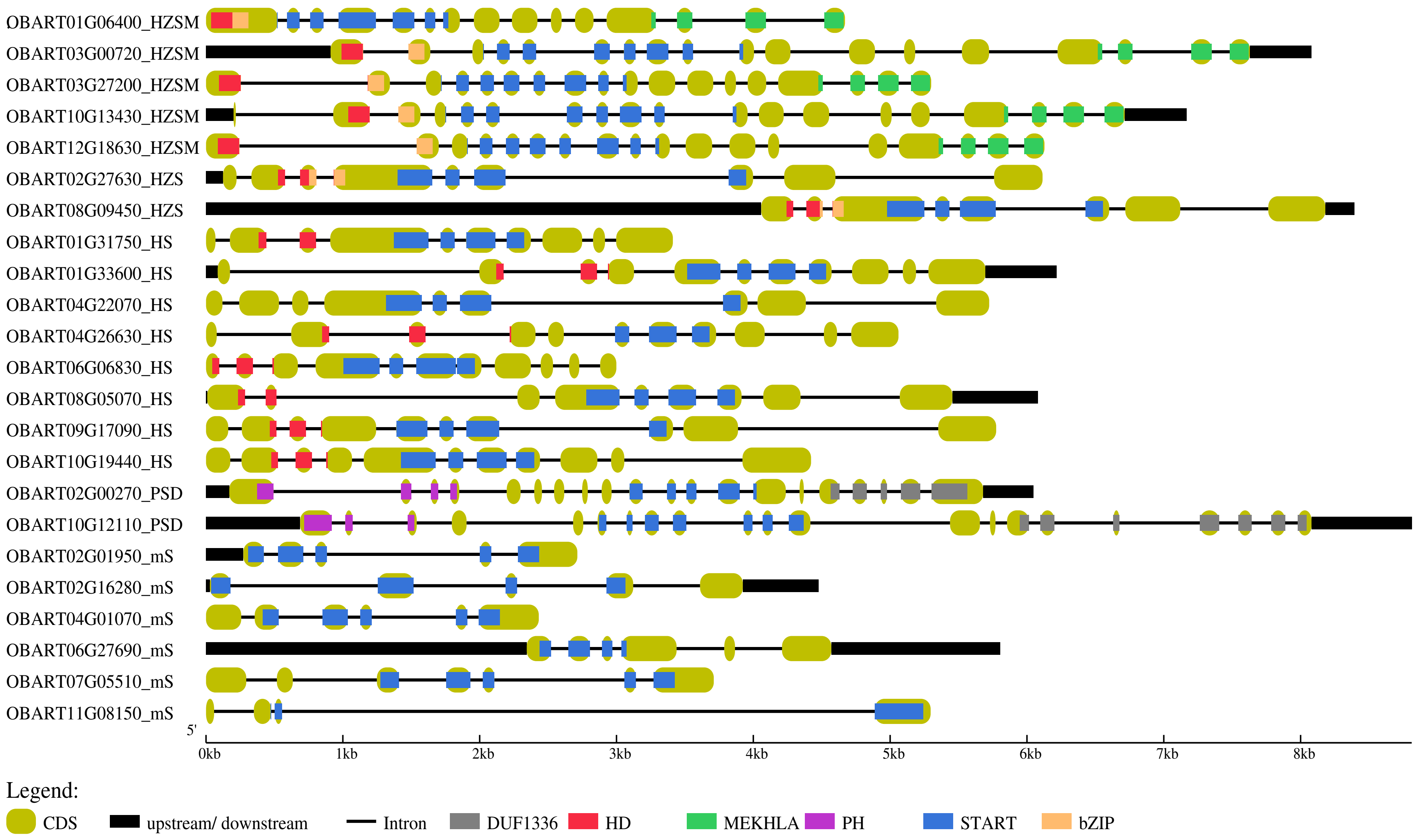

Supplementary Figure 1F. Gene structure analysis of the twenty-three STARTs in *Oryza barthii*: exon-intron patterns

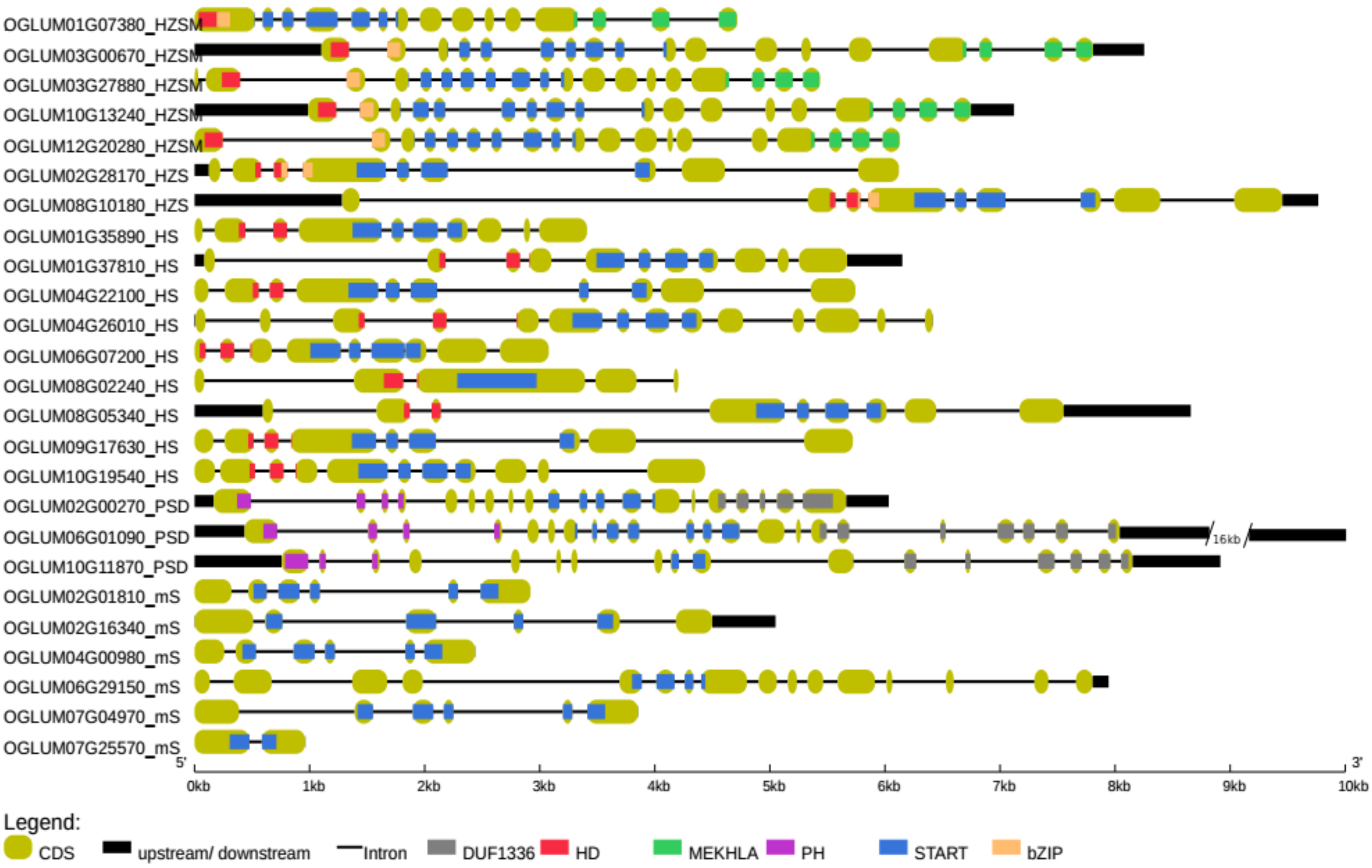

Supplementary Figure 1G. Gene structure analysis of the twenty-five STARTs in *Oryza glumipatula*: exon-intron patterns

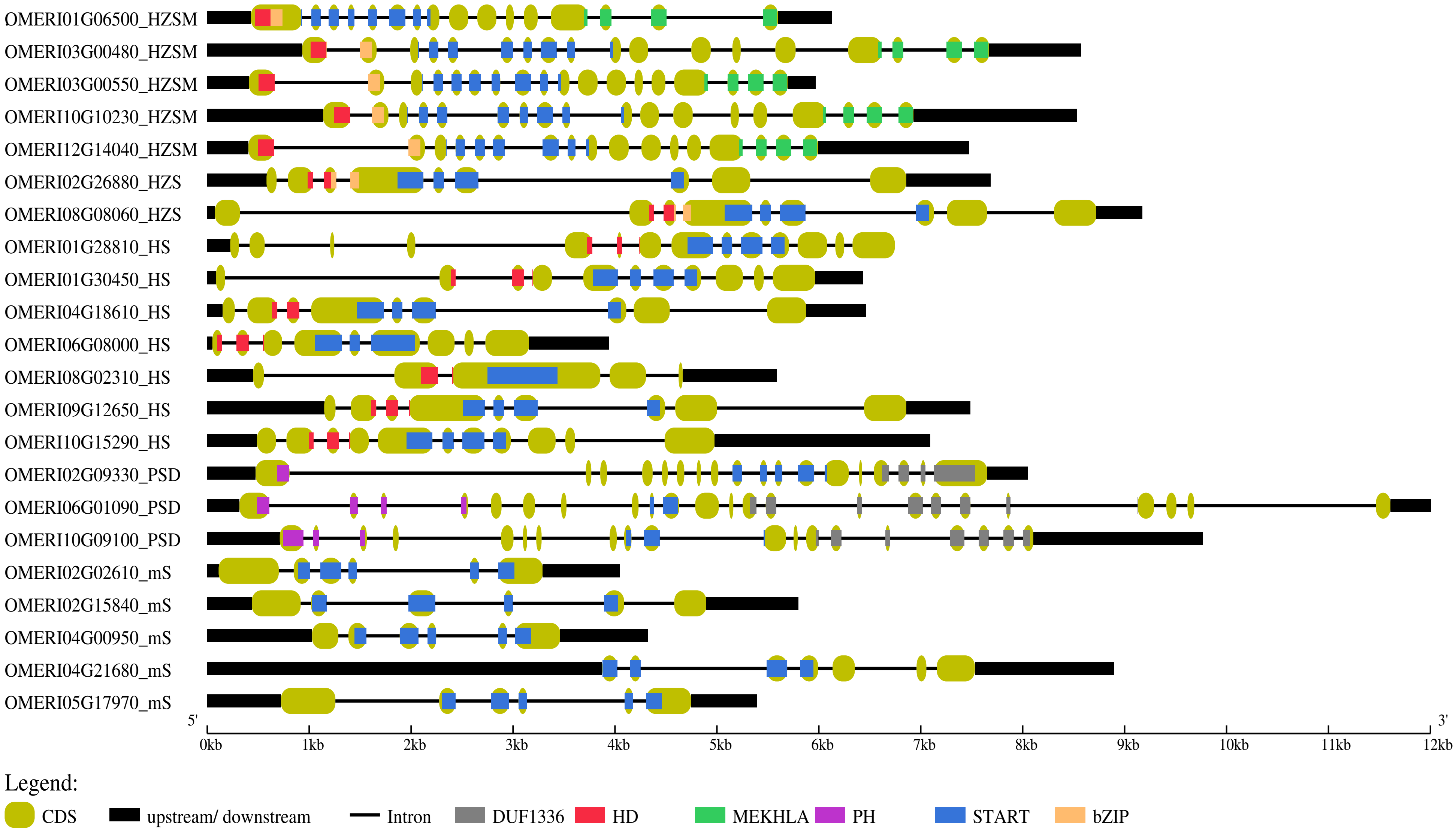

Supplementary Figure 1H. Gene structure analysis of the twenty-two STARTs in *Oryza meridionalis*: exon-intron patterns

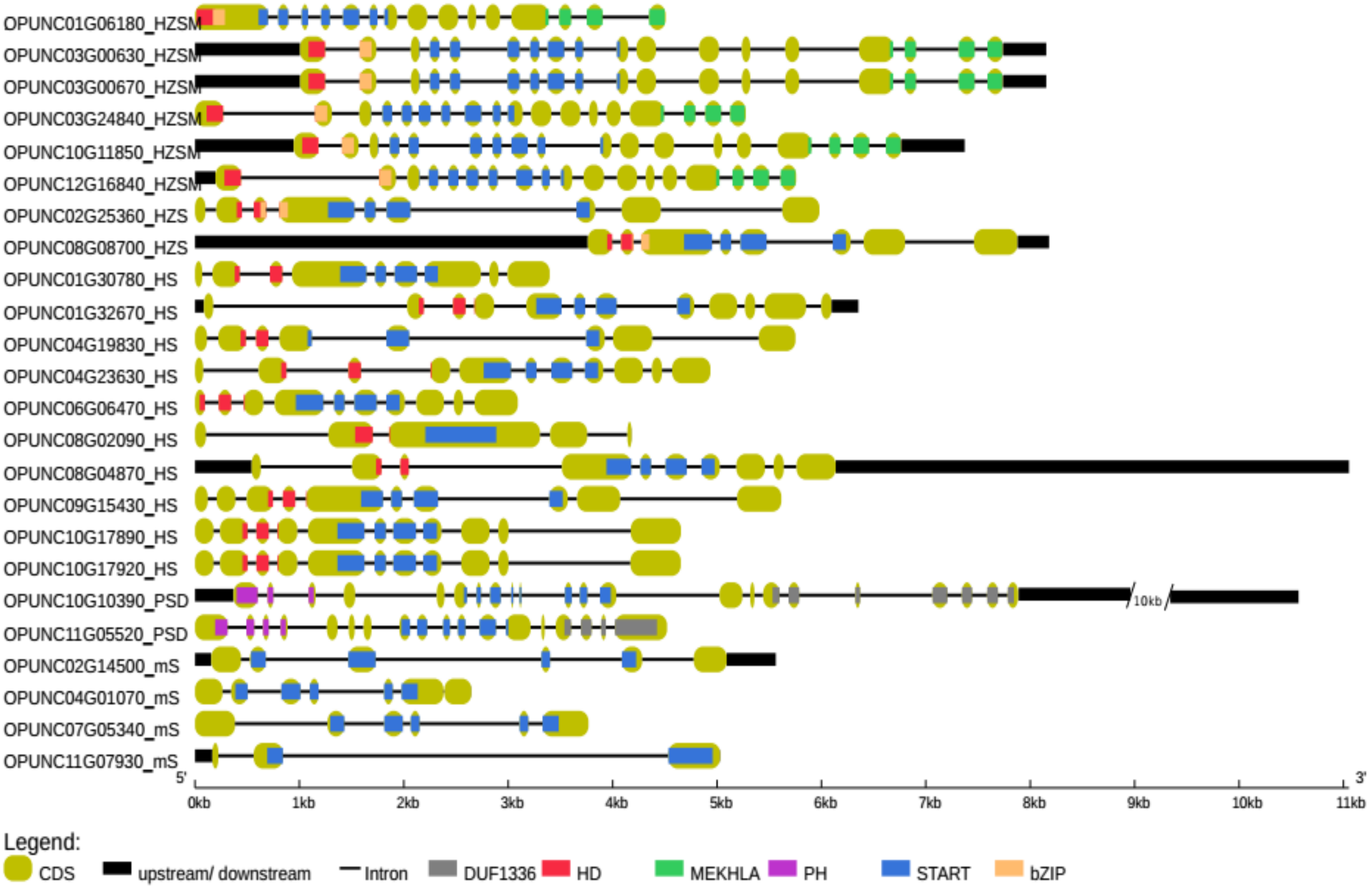

Supplementary Figure 1I. Gene structure analysis of the twenty-four STARTs in *Oryza punctata*: exon-intron patterns

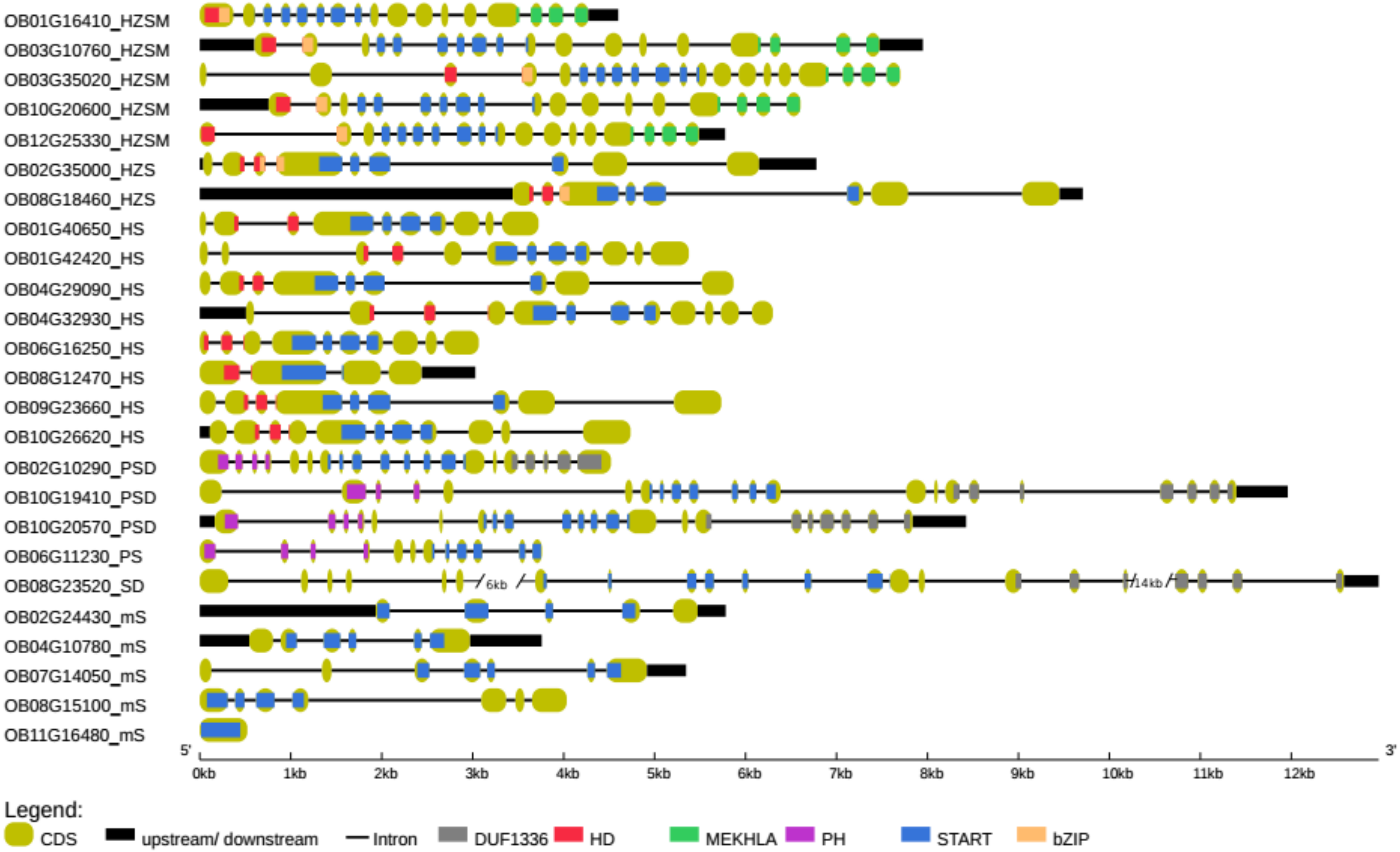

Supplementary Figure 1J. Gene structure analysis of the twenty-five STARTs in *Oryza brachyantha*: exon-intron patterns
