# Supplementary material for: StAR-Related Lipid Transfer (START) Domains Across the Rice Pangenome Reveal How Ontogeny Recapitulated Selection Pressures During Rice Domestication
Source: Front Genet. 2021 Sep 8;12:737194. doi: 10.3389/fgene.2021.737194 (PMC8455945; doi:10.3389/fgene.2021.737194)
Supplement: Supplementary Figure 2 — Collinear blocks for the 10 rice genomes (A) Oryza sativa var. japonica, (B) Oryza sativa var. indica, (C) Oryza glaberrima, (D) Oryza rufipogon, (E) Oryza nivara, (F) Oryza barthii, (G) Oryza glumaepatula, (H) Oryza meridionalis, (I) Oryza punctata, and (J) Oryza brachyantha. [file Data_Sheet_2.PDF]

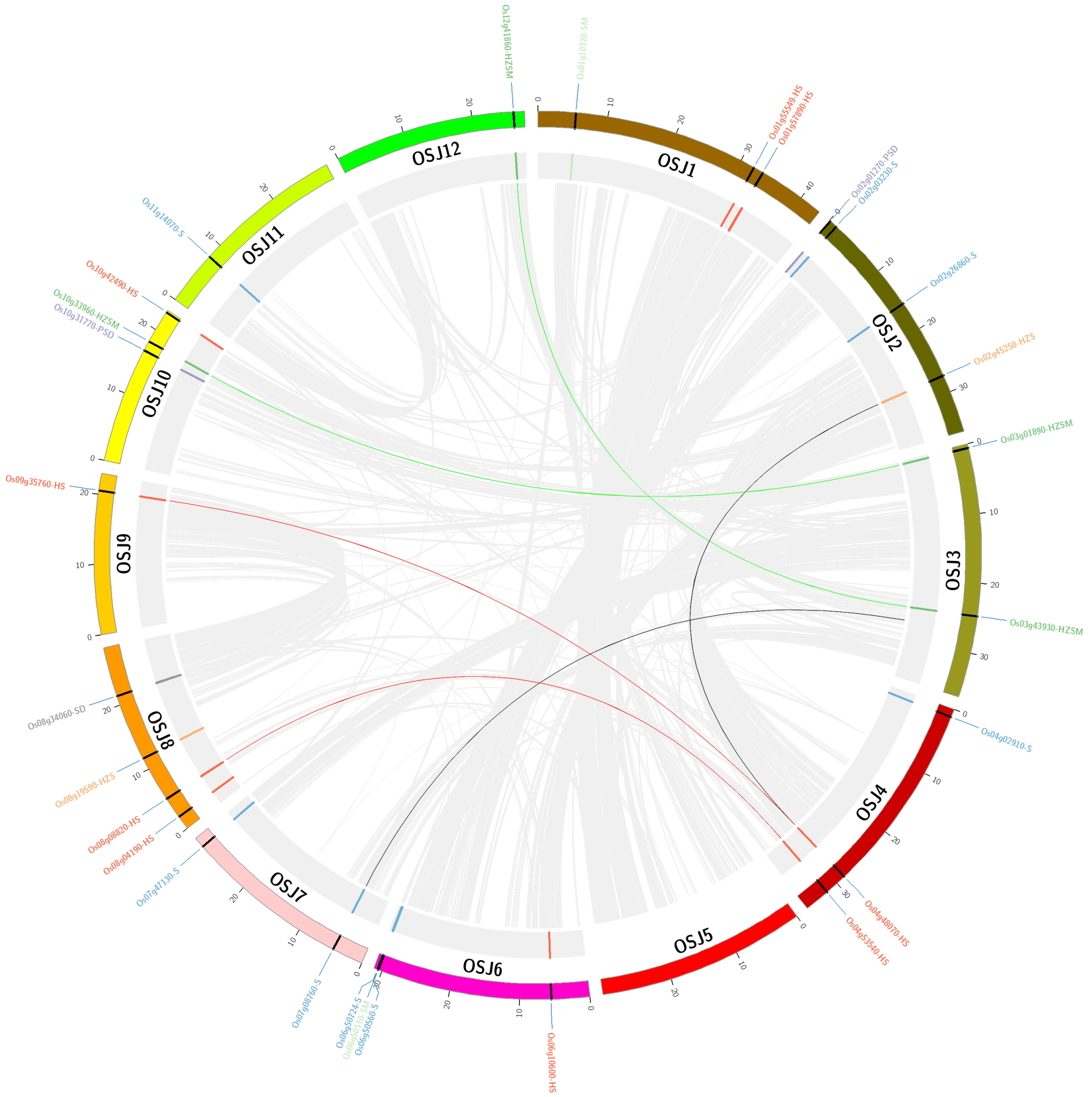

Supplementary Figure 2A. Collinear blocks of the *Oryza sativa var. japonica* genome.

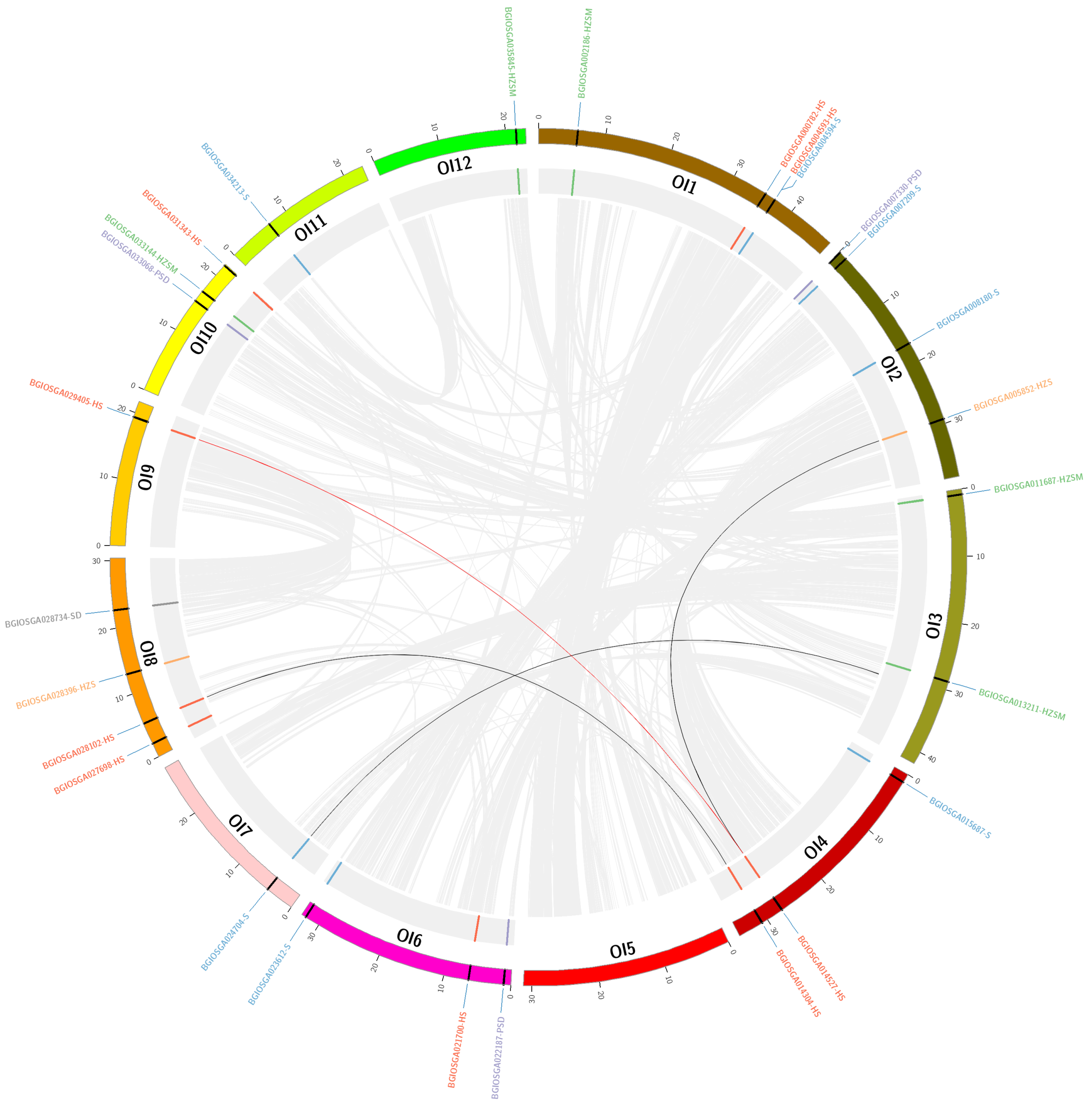

Supplementary Figure 2B. Collinear blocks of the *Oryza sativa var. indica* genome.

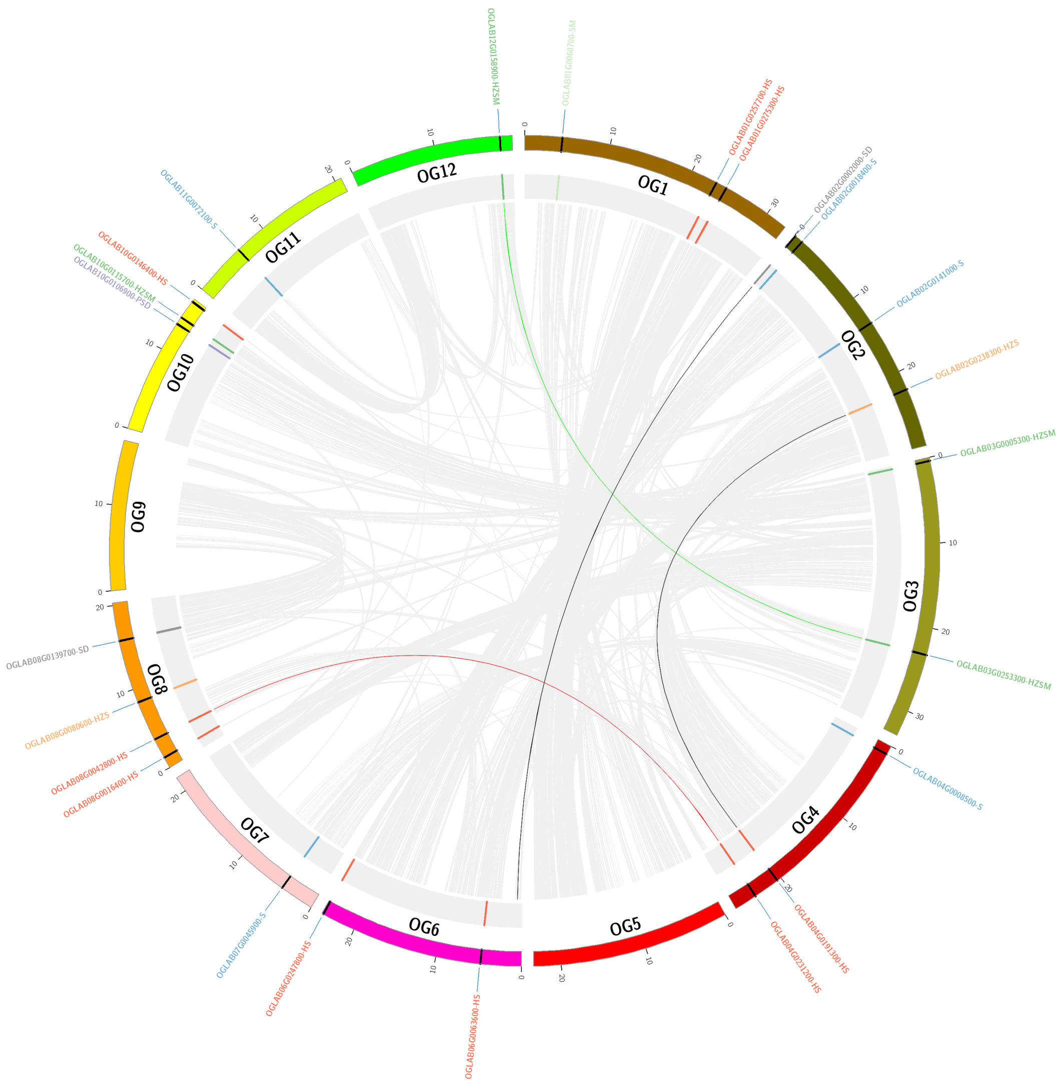

Supplementary Figure 2C. Collinear blocks of the *Oryza glaberrima* genome.

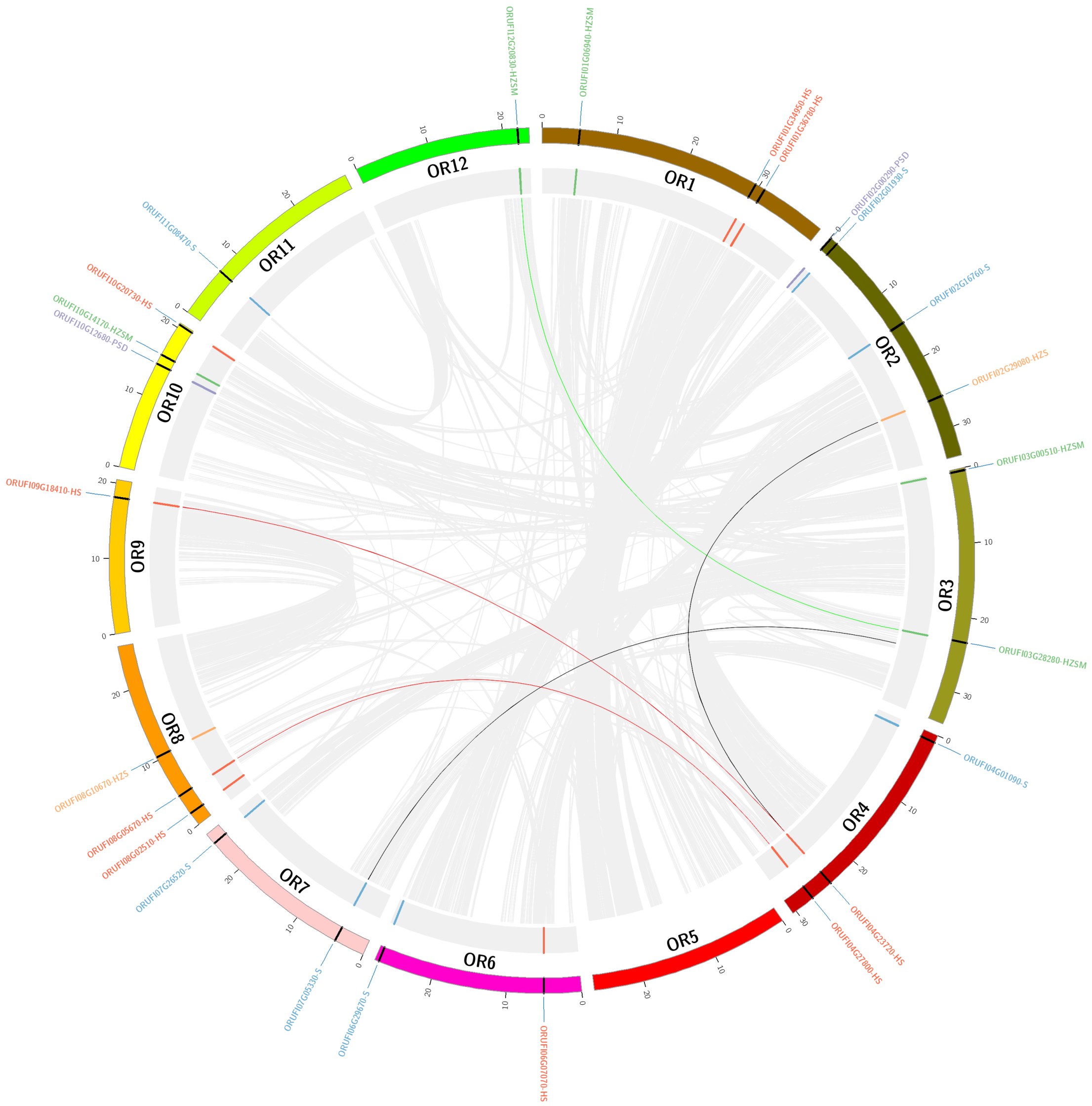

Supplementary Figure 2D. Collinear blocks of the *Oryza rufipogon* genome.

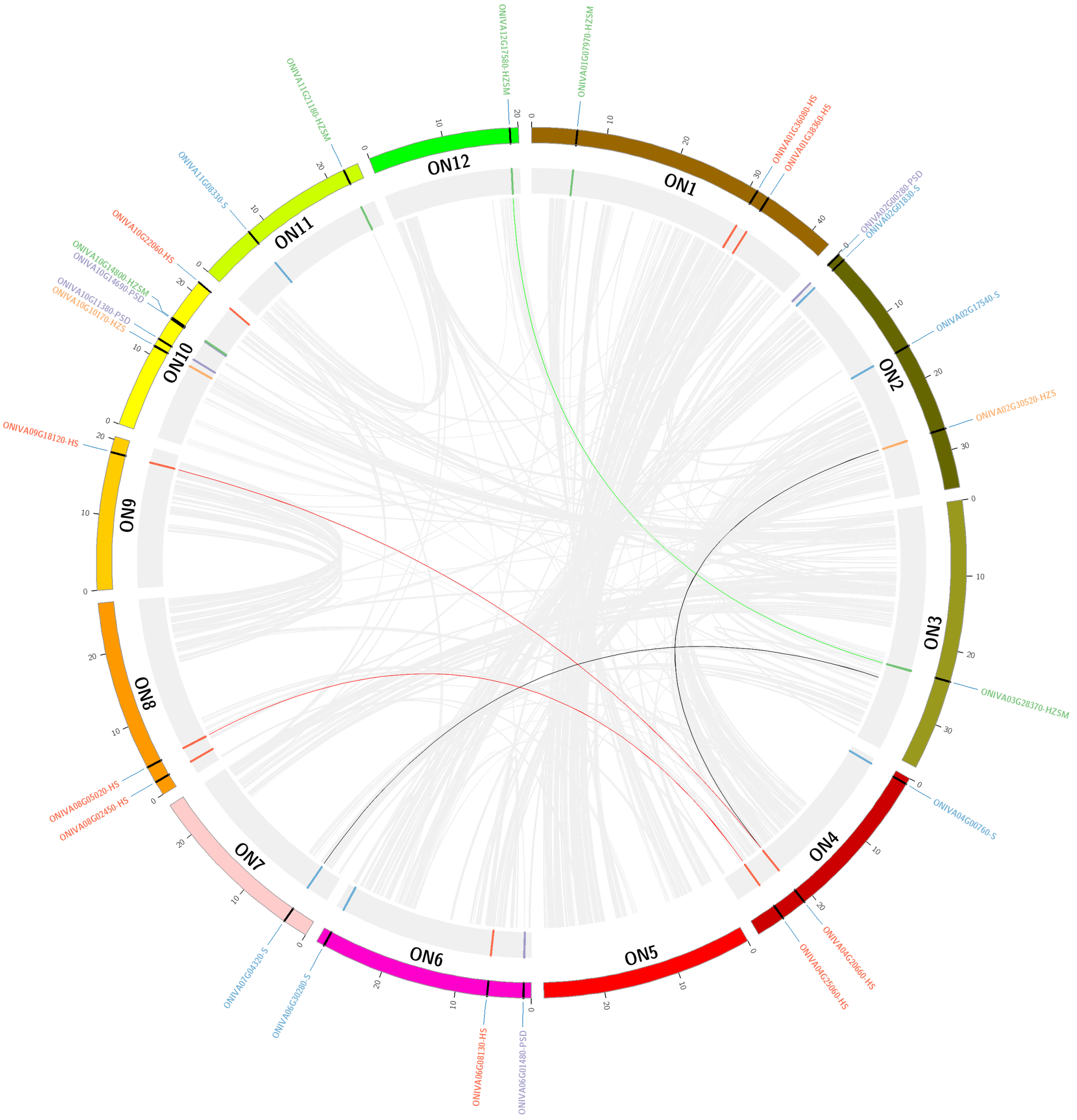

Supplementary Figure 2E. Collinear blocks of the *Oryza nivara* genome.

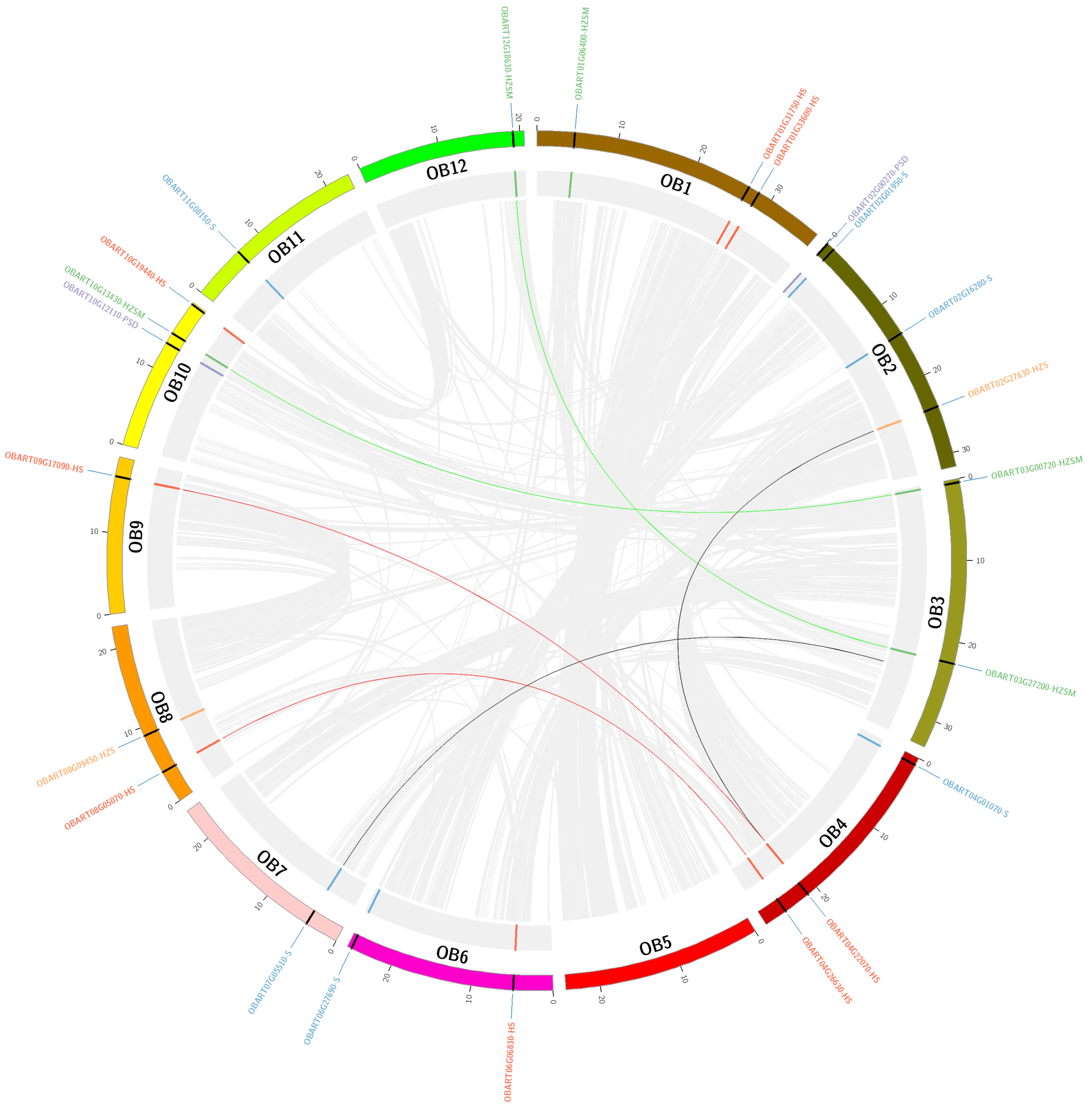

Supplementary Figure 2F. Collinear blocks of the *Oryza barthii* genome

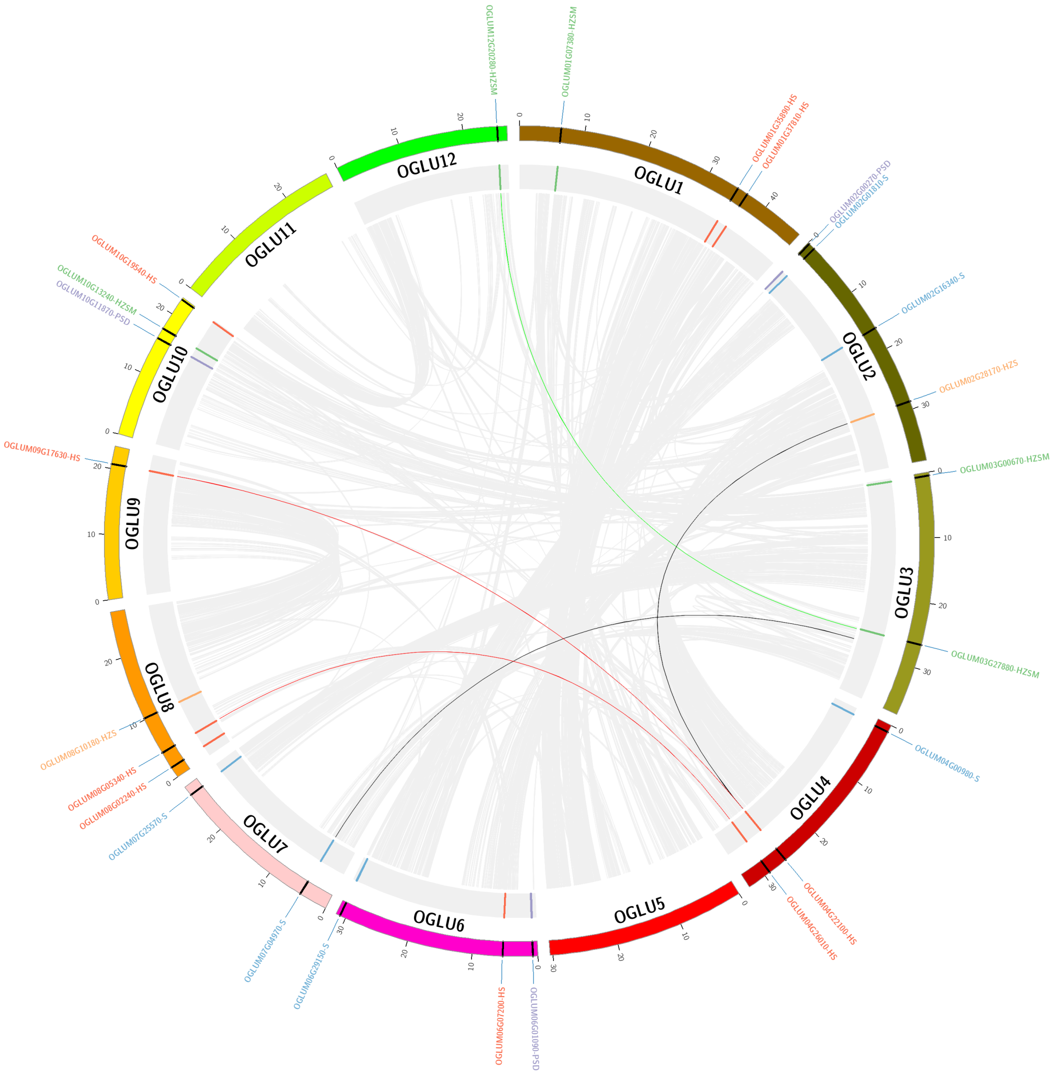

Supplementary Figure 2G. Collinear blocks of the *Oryza glumaepatula* genome.

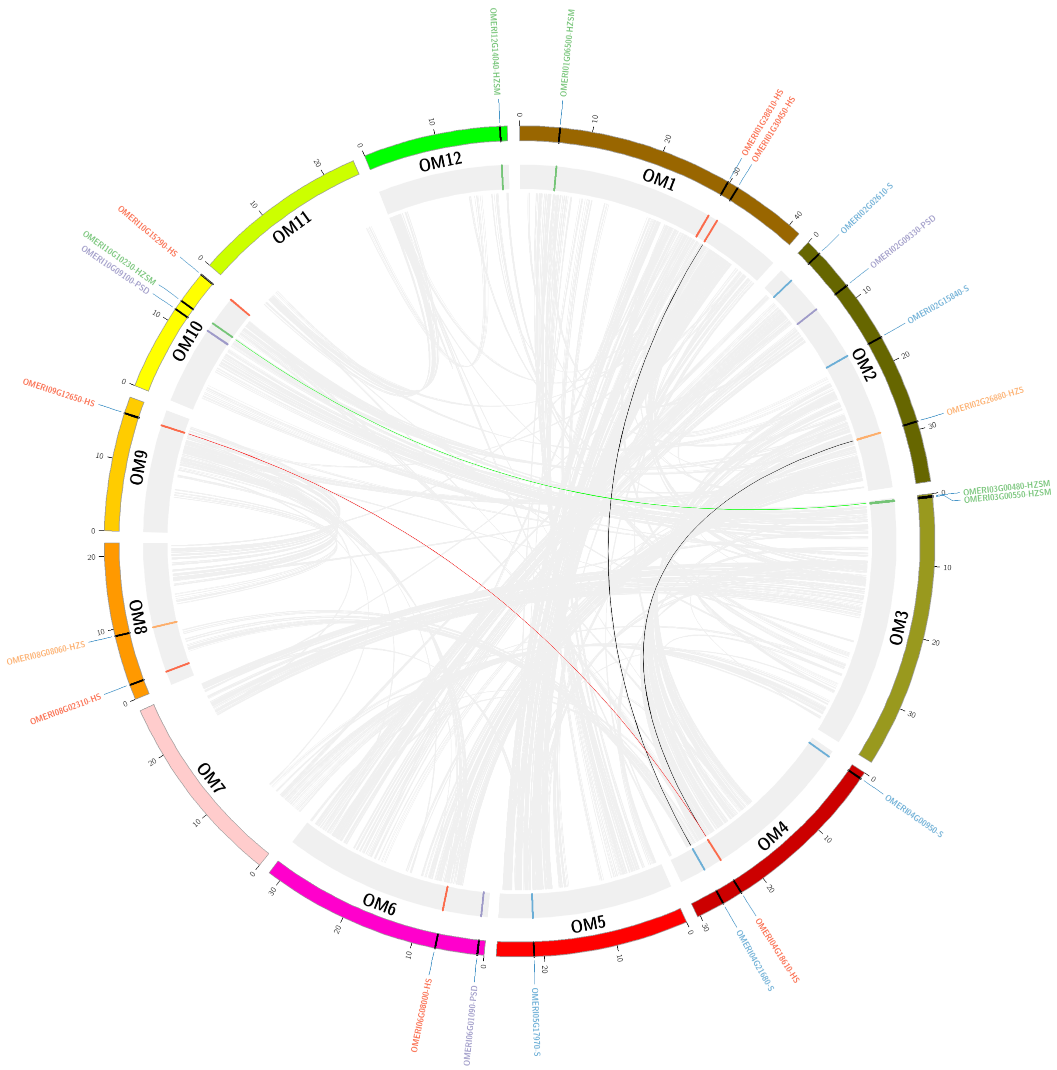

Supplementary Figure 2H. Collinear blocks of the *Oryza meridionalis* genome.

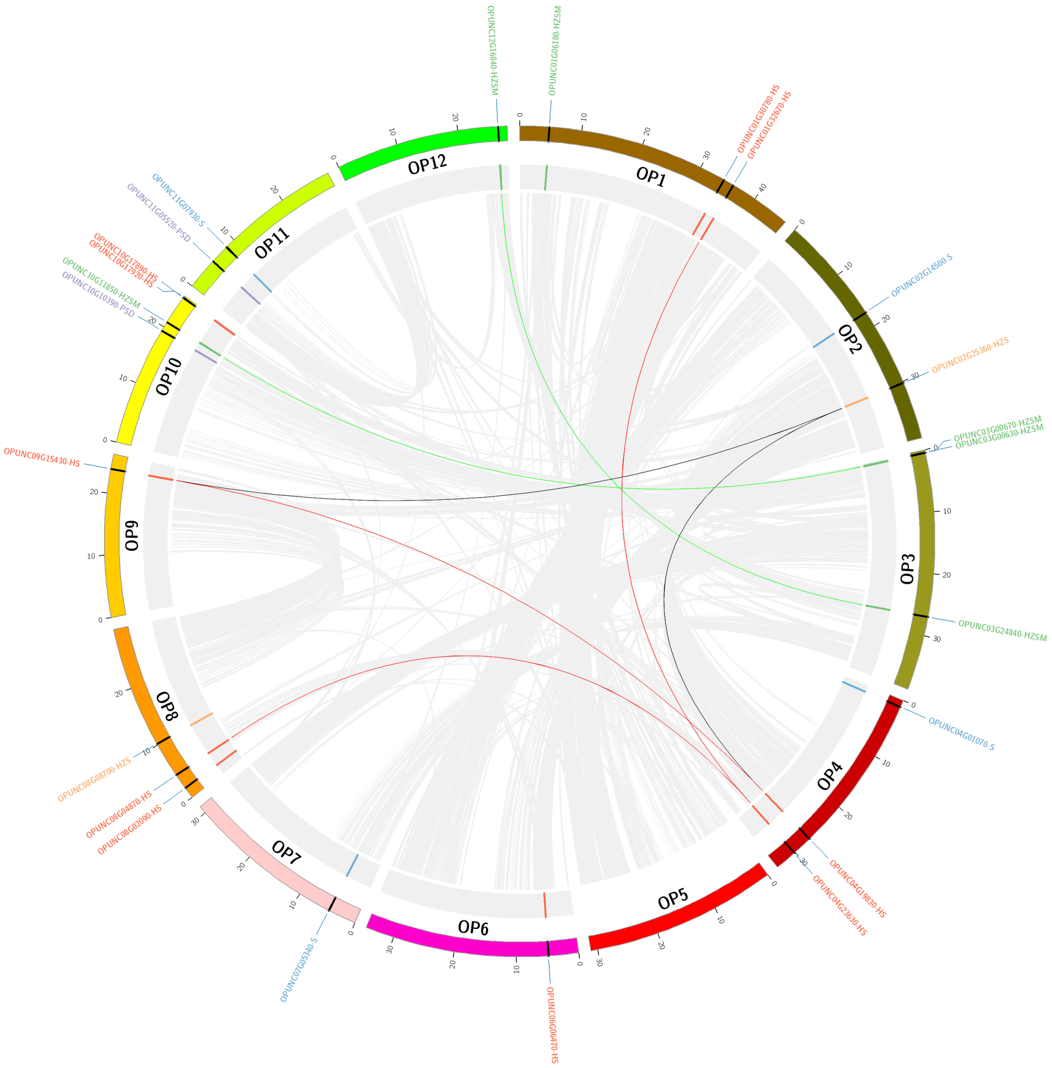

Supplementary Figure 2I. Collinear blocks of the *Oryza punctata* genome.

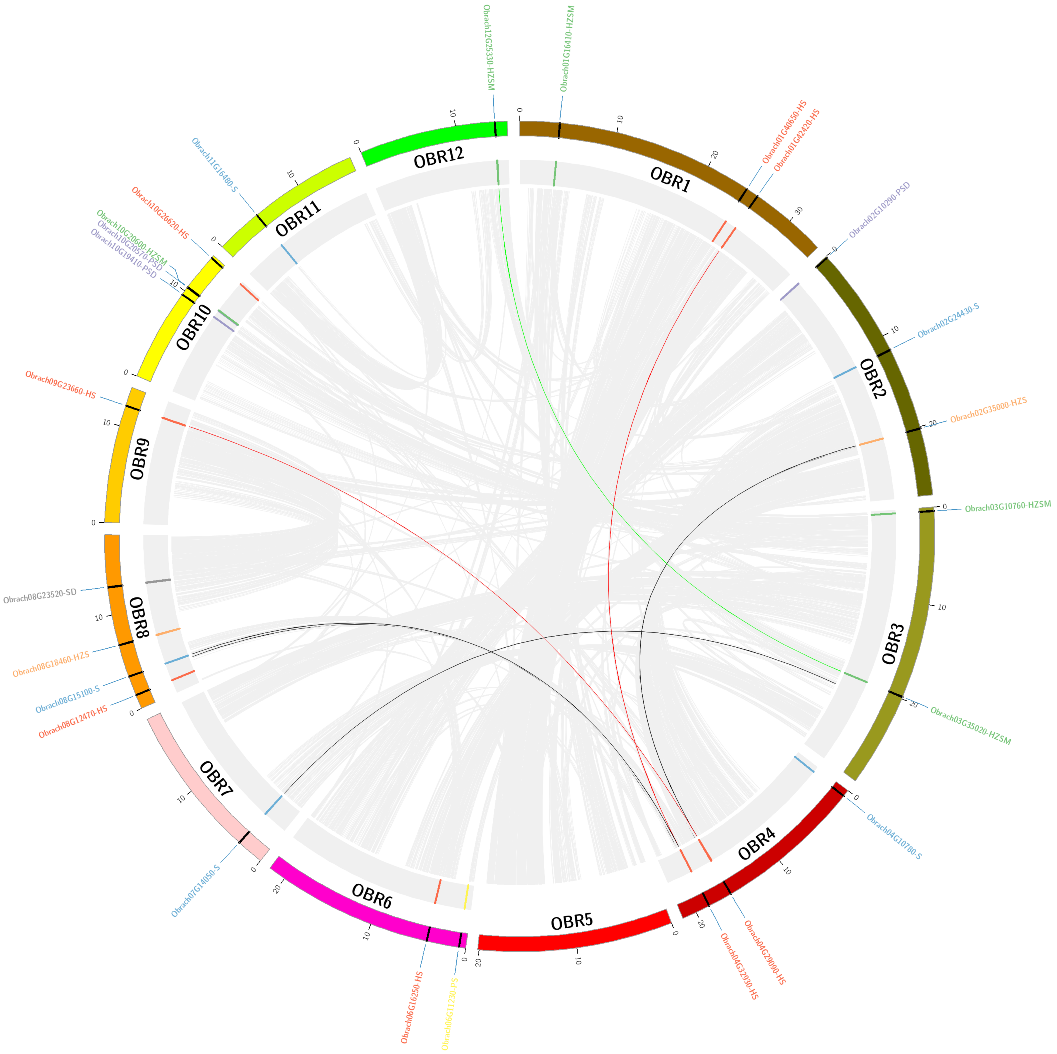

Supplementary Figure 2J. Collinear blocks of the *Oryza brachyantha* genome.
