# Supplementary material for: StAR-Related Lipid Transfer (START) Domains Across the Rice Pangenome Reveal How Ontogeny Recapitulated Selection Pressures During Rice Domestication
Source: Front Genet. 2021 Sep 8;12:737194. doi: 10.3389/fgene.2021.737194 (PMC8455945; doi:10.3389/fgene.2021.737194)
Supplement: Supplementary Figure 3 — (A) Ka, (B) Ks, and (C) Ka/Ks values for START homologs of different Oryza species with respect to Oryza sativa var. japonica. [file Data_Sheet_3.PDF]

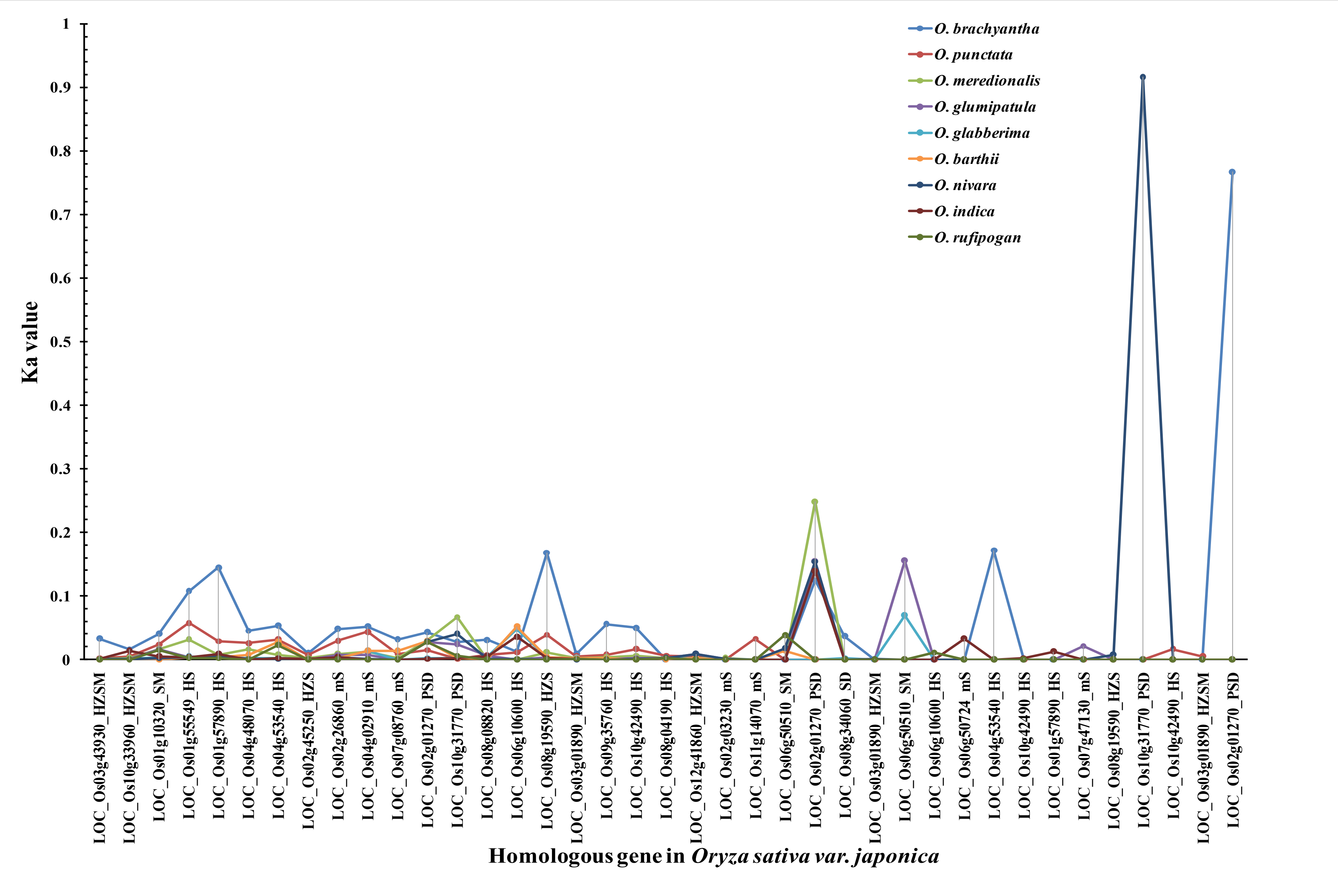

Supplementary Figure 3A. Ka values for START homologues of different Oryza species with respect to *Oryza sativa var. japonica*

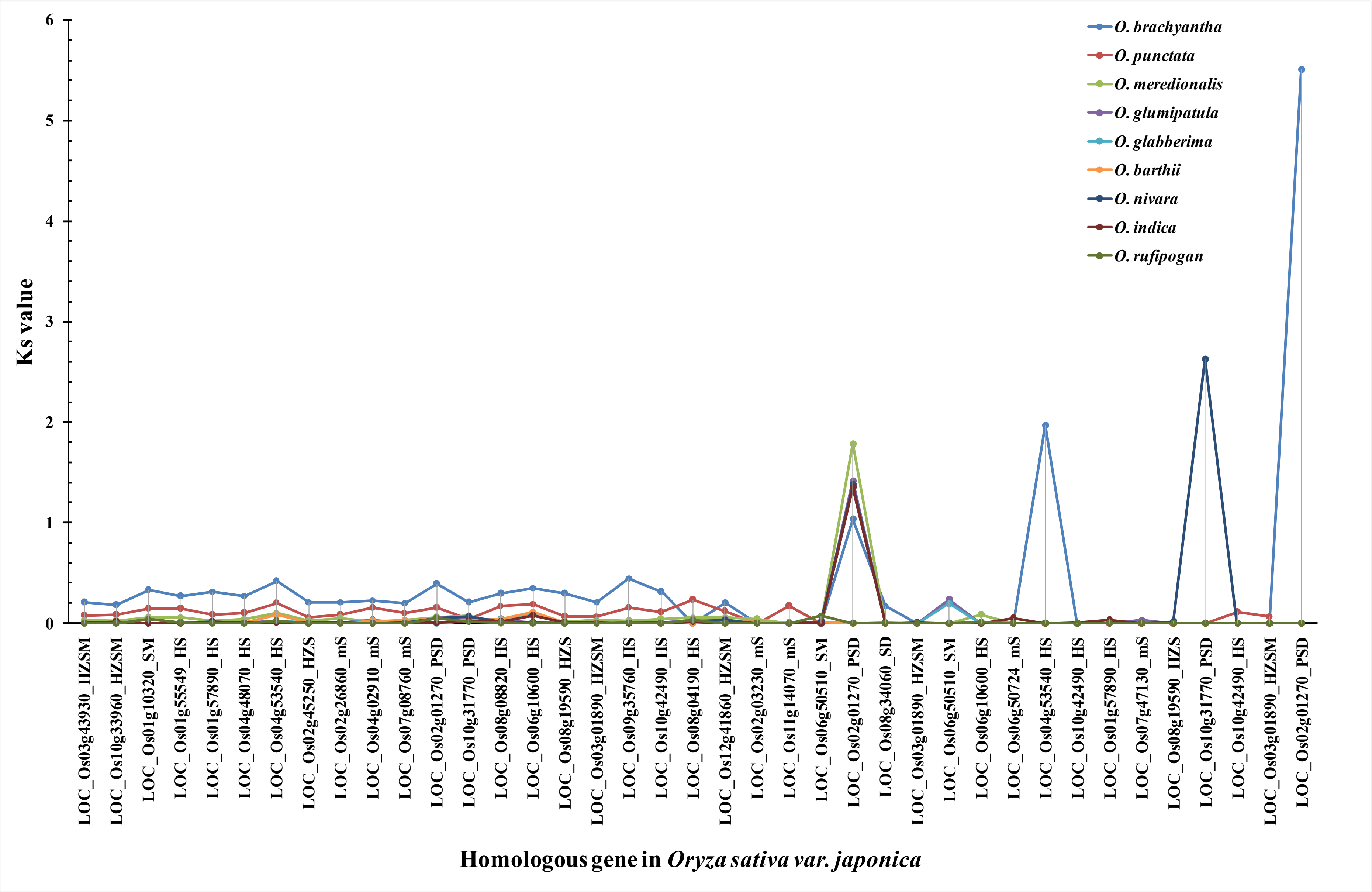

Supplementary Figure 3B. Ks values for START homologues of different Oryza species with respect to *Oryza sativa var. japonica*

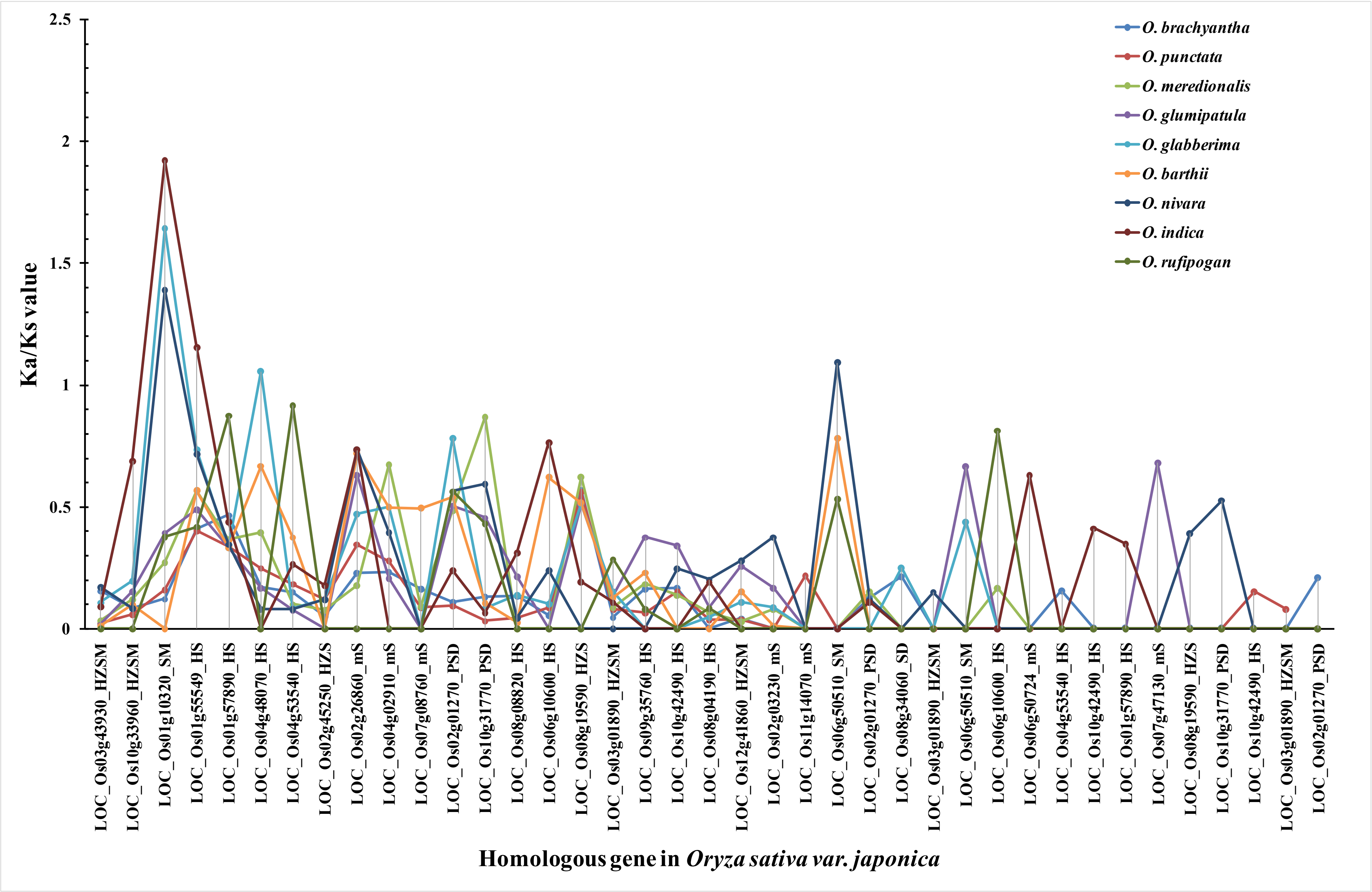

Supplementary Figure 3C. Ka/Ks values for START homologues of different Oryza species with respect to *Oryza sativa var. japonica*
