# Supplementary material for: StAR-Related Lipid Transfer (START) Domains Across the Rice Pangenome Reveal How Ontogeny Recapitulated Selection Pressures During Rice Domestication
Source: Front Genet. 2021 Sep 8;12:737194. doi: 10.3389/fgene.2021.737194 (PMC8455945; doi:10.3389/fgene.2021.737194)
Supplement: Supplementary Figure 4 — Conditional gene expression pattern of START genes for (A) different developmental stages (B) various anatomical parts. [file Data_Sheet_4.PDF]

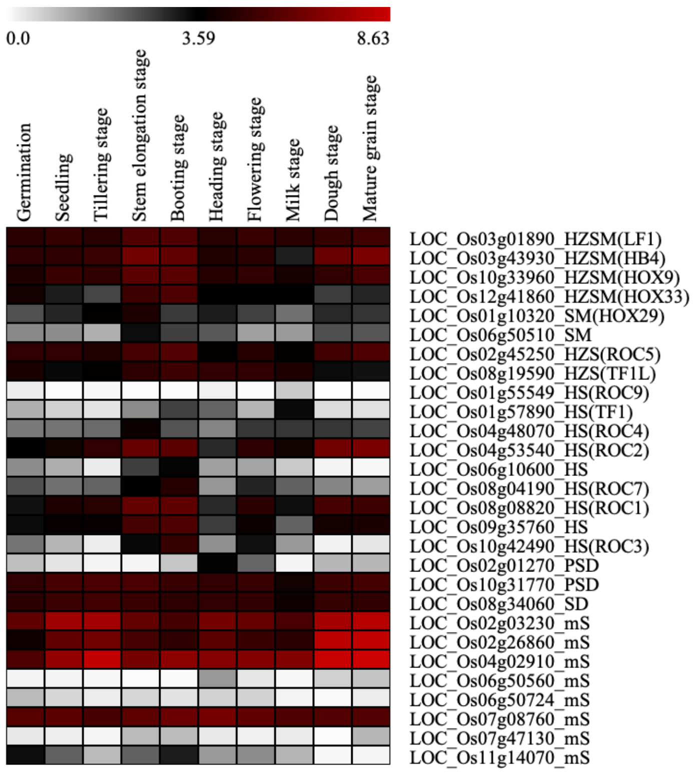


**Supplementary Figure 4A.** Conditional gene expression pattern of START genes at different developmental Stages in *Oryza sativa var. japonica*

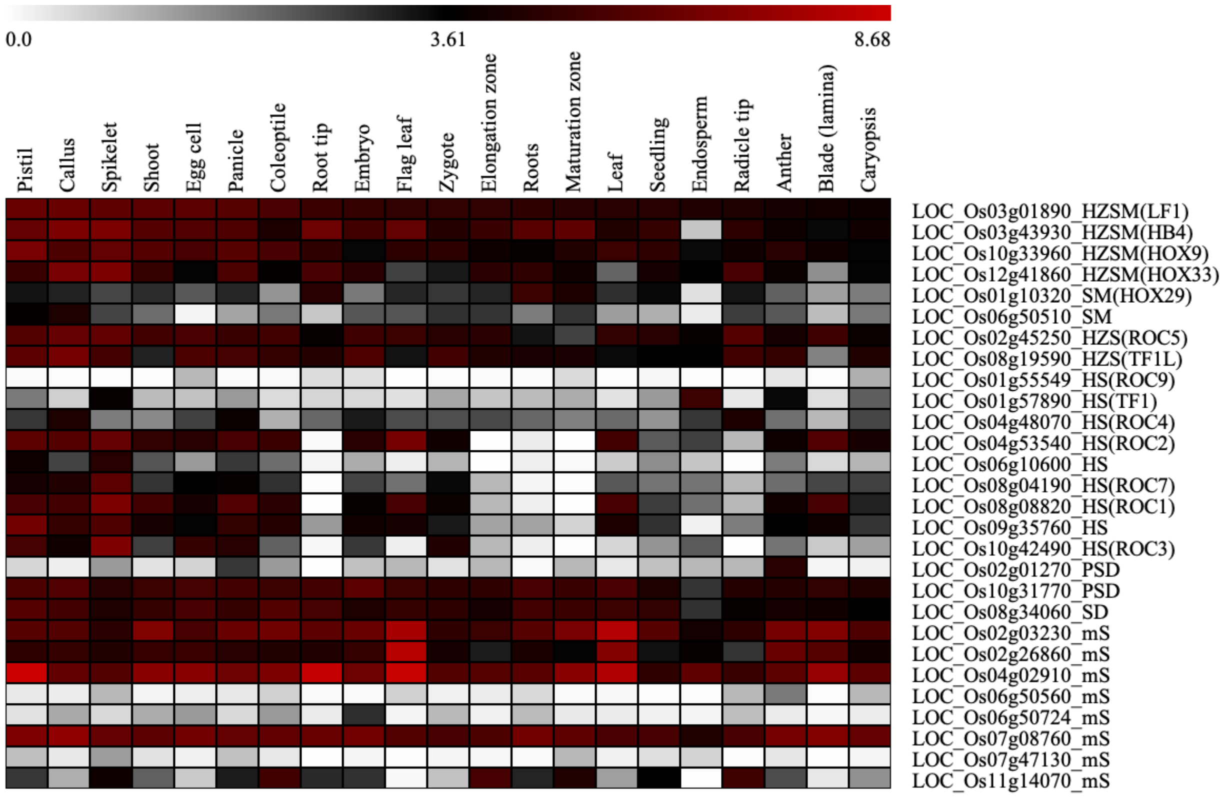


**Supplementary Figure 4B.** Conditional gene expression pattern of START genes in various anatomical parts in *Oryza sativa var. japonica*
